# In vivo human T cell engineering with enveloped delivery vehicles

**DOI:** 10.1038/s41587-023-02085-z

**Published:** 2024-01-11

**Authors:** Jennifer R. Hamilton, Evelyn Chen, Barbara S. Perez, Cindy R. Sandoval Espinoza, Min Hyung Kang, Marena Trinidad, Wayne Ngo, Jennifer A. Doudna

**Affiliations:** 1 Department of Molecular and Cell Biology, University of California, Berkeley, Berkeley, CA USA; 2 Innovative Genomics Institute, University of California, Berkeley, CA USA; 3 Gladstone Institutes, San Francisco, CA USA; 4 California Institute for Quantitative Biosciences, University of California, Berkeley, Berkeley, CA USA; 5 Howard Hughes Medical Institute, University of California, Berkeley, Berkeley, USA; 6 Gladstone-UCSF Institute of Genomic Immunology, San Francisco, CA USA; 7 Molecular Biophysics and Integrated Bioimaging Division, Lawrence Berkeley National Laboratory, Berkeley, CA USA; 8 Department of Chemistry, University of California, Berkeley, Berkeley, CA USA; 9 Present Address: Azalea Therapeutics, Berkeley, CA USA

**Keywords:** Protein delivery, Genetic vectors, Genetic engineering, Immunotherapy

## Abstract

Viruses and virally derived particles have the intrinsic capacity to deliver molecules to cells, but the difficulty of readily altering cell-type selectivity has hindered their use for therapeutic delivery. Here, we show that cell surface marker recognition by antibody fragments displayed on membrane-derived particles encapsulating CRISPR–Cas9 protein and guide RNA can deliver genome editing tools to specific cells. Compared to conventional vectors like adeno-associated virus that rely on evolved capsid tropisms to deliver virally encoded cargo, these Cas9-packaging enveloped delivery vehicles (Cas9-EDVs) leverage predictable antibody–antigen interactions to transiently deliver genome editing machinery selectively to cells of interest. Antibody-targeted Cas9-EDVs preferentially confer genome editing in cognate target cells over bystander cells in mixed populations, both ex vivo and in vivo. By using multiplexed targeting molecules to direct delivery to human T cells, Cas9-EDVs enable the generation of genome-edited chimeric antigen receptor T cells in humanized mice, establishing a programmable delivery modality with the potential for widespread therapeutic utility.

## Abstract (web summary)

Cell-specific molecular delivery with enveloped delivery vehicles enables genome editing ex vivo and in vivo.

## Main

Therapeutic interventions involving genome editing require the safe and effective delivery of molecules into target cell nuclei^1–3^. Although such capability would transform both clinical and research applications, current non-viral delivery is limited to cells treated ex vivo^4–6^, tissues targeted by local administration^7,8^ or the liver because of its natural propensity for molecular uptake^8,9^. Recent lipid nanoparticle formulations have been described with tropism for non-hepatic cells or organs^10,11^, but expansion of in vivo genome editing applications will probably require multiple approaches for molecular delivery to specific cells or organs inside the body following systemic administration.

Retargeting the tropism of viruses or viral vectors is an established delivery strategy involving the surface display of a cell-selective targeting molecule alongside a viral glycoprotein required for cell entry by fusion at the plasma membrane or in the low-pH environment of the endosome^12–15^. Recent progress leverages a mutant form of the vesicular stomatitis virus glycoprotein (VSVG), VSVGmut, that maintains endosomal fusion activity but lacks native low-density lipoprotein receptor binding affinity^16–18^. Pairing VSVGmut with cell-specific targeting molecules can redirect lentiviral transgene delivery and has enabled high-throughput screening of T cell and B cell receptor libraries to study receptor–antigen interactions^19,20^.

Particles cloaked in cellular membrane fragments—such as retrovirus-like particles (VLPs), extracellular vesicles and biomimetic nanoparticles—are gaining in popularity for the delivery of molecular cargo. For this class of EDVs, bioengineering is required to achieve molecular cargo packaging and control of targeting and fusogenic activity. Here, we show that human cell-specific genome editing can be achieved both ex vivo and in vivo by pairing the display of VSVGmut with antibody-derived single-chain variable fragments (scFvs) on EDVs that package Cas9 ribonucleoprotein (RNP) complexes (Cas9-EDVs). EDVs described in this paper leverage retroviral VLP assembly for the transient delivery of Cas9 RNPs^8,21–27^. We find that Cas9-EDVs achieve targeted genome editing within in vivo-generated chimeric antigen receptor (CAR) T cells in mice with a humanized immune system, with no off-target delivery to liver hepatocytes. These data show that EDVs are a programmable platform for delivering molecular cargo to specific cell types for complex genome engineering—both gene delivery and targeted gene disruption—inside the body.

## Results

### Receptor-mediated delivery and genome editing with Cas9-EDVs

**Fig. 1:**
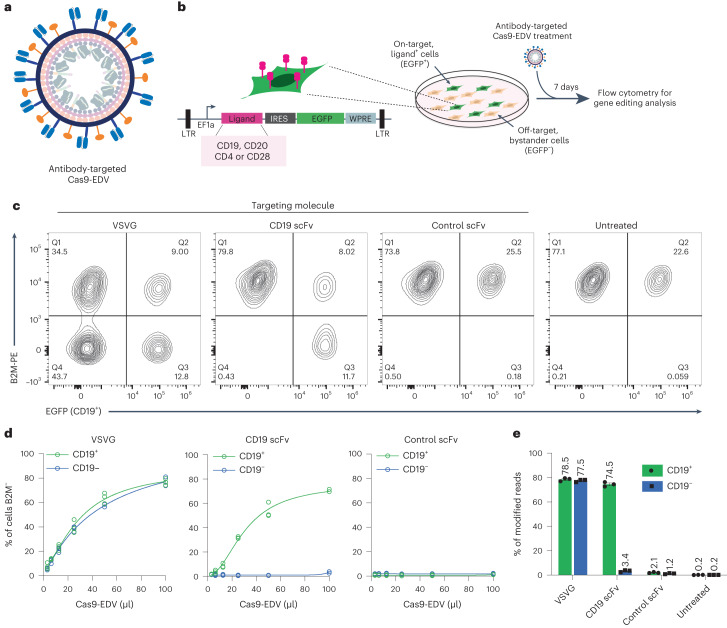
Cell-specific genome editing with antibody-targeted Cas9-EDVs. **a**, Schematic scFv targeting molecules (blue) and VSVGmut (orange) on the exterior surface of a Cas9-EDV. Cas9-EDVs package pre-formed Cas9-sgRNA complexes to avoid genetically encoding genome editors within a viral genome. **b**, Experimental outline and schematic of the lentiviral vector used for engineering HEK293T EGFP cells that express heterologous ligands on the plasma membrane (for example, CD19). To promote cellular engineering by single lentiviral integration events, engineered cell mixtures were generated through low multiplicity of infection to achieve <25% EGFP^+^ cells. Engineered cell mixtures were challenged with *B2M*-targeting Cas9-EDVs to test targeting molecule activity. **c**–**e**, Assessment of antibody-targeted Cas9-EDV activity. HEK293T and CD19 EGFP HEK293T cells were mixed at an approximate ratio of 3:1 and treated with *B2M*-targeting Cas9-EDVs displaying various targeting molecule pseudotypes. Cas9-EDVs were concentrated 10× and cells were treated with 50 μl Cas9-EDVs (**c**,**e**) or in a dilution curve (**d**). Analysis was performed 7 days post treatment to assess *B2M* knockout in EGFP^+^ (on-target) and EGFP^−^ (bystander) cells by flow cytometry (**c**,**d**) and amplicon sequencing (**e**). *n* = 3 technical replicates were used in all experiments except for the 100 μl dose of CD19-scFv in **d** (*n* = 2). Individual replicate values and four-parameter non-linear regression curves are plotted in **d**. Error bars in **e**, s.e.m. Source data

A major challenge for in vivo delivery of editing enzymes is the lack of vehicles capable of targeting specific cell types. VLPs can package Cas9 RNP complexes produced by over-expressing Cas9 fused to the carboxy-terminal end of the viral Gag polyprotein during VLP production, but cell-selective VLP targeting has relied on cell infection strategies evolved by enveloped viruses^22^. To test whether VLPs could be reformulated as programmable EDVs, we first cloned a CD19 targeting antibody as an scFv fused to the stalk and transmembrane domain of CD8*a*, a strategy commonly used in CAR architecture^28^ (Fig. 1a and Supplementary Fig. 1a,b). Given that Cas9-VLPs bud from the plasma membrane of transfected producer cells, we reasoned that co-expression of the scFv fusion and VSVGmut together with lentiviral components that are necessary for Cas9 RNP encapsulation would generate Cas9-EDVs possessing both receptor specificity and endosomal escape capability, respectively.

To analyze the receptor-mediated function of Cas9-EDVs, we generated a HEK293T cell line that co-expresses both the B cell ligand CD19 and EGFP, enabling assessment of genome editing in on-target EGFP^+^ cells and off-target EGFP^−^ bystander cells (Fig. 1b). We produced Cas9-EDVs containing single guide RNA (sgRNA) targeting the β-2 microglobulin (*B2M*) gene and outwardly displaying either VSVG, CD19 scFv+VSVGmut or a control scFv+VSVGmut that should not recognize the target cells in this experiment. In an ~3:1 mixture of HEK293T and CD19 EGFP 293T cells, the VSVG Cas9-EDVs mediated genome editing in both CD19^+^ and CD19^−^ populations, whereas the CD19-scFv Cas9-EDVs induced the knockout of *B2M* only in CD19^+^ cells (Fig. 1c). No *B2M* knockout was observed in either the CD19^+^ or CD19^−^ cells using the control scFv+VSVGmut Cas9-EDVs. Antibody-targeted Cas9-EDV activity was titratable, with up to 74% of target cells exhibiting *B2M* knockout and little to no editing detected in bystander, non-target cells (Fig. 1d,e). Antibody-targeted Cas9-EDVs produced genome edits in target cells present at 2–92% of a cell mixture, whereas bystander cell editing was unchanged (Supplementary Fig. 1c). Together, these results demonstrate the ability of EDVs to deliver functional molecular cargo in a receptor-mediated fashion.

### Programmable cell-specific genome editing with Cas9-EDVs

Receptor-mediated delivery of genome editing molecules could enable targeted engineering of any cell type as a function of its surface antigens. To test this possibility, we investigated the modularity and programmability of Cas9-EDVs to direct genome editing in HEK293T cells displaying various plasma membrane proteins normally expressed by human immune cells, including CD20, CD4 and CD28 (Fig. 1b and Supplementary Fig. 2a). Cas9-EDVs displaying VSVGmut and scFv-based CD20, CD4 and CD28 targeting molecules, generated in both variable heavy (VH)–linker–variable light (VL) and VL–linker–VH orientations (Supplementary Table 1)^29^, induced up to 80% genome editing that was titratable and selective for ligand^+^ over ligand^−^ cells (Supplementary Fig. 2b). Not all scFvs produced the same level of on-target cell editing, but in no case was an scFv-displaying EDV able to induce more than minimal editing in off-target cells lacking the cognate surface receptor protein. Furthermore, in all engineered cell mixtures, ligand^+^ and ligand^−^ cells were similarly susceptible to genome editing when treated with control Cas9-EDVs that express the VSVG fusogen (Supplementary Fig. 2b). A panel of CD19, CD20 and CD4 antibody-targeted EDVs only mediated genome editing in cells expressing their matched ligand and not in mismatched ligand-expressing cells, demonstrating that delivery requires antibody–antigen interactions (Supplementary Fig. 2c).

### Cas9-EDV optimization and study of nonessential components

**Fig. 2:**
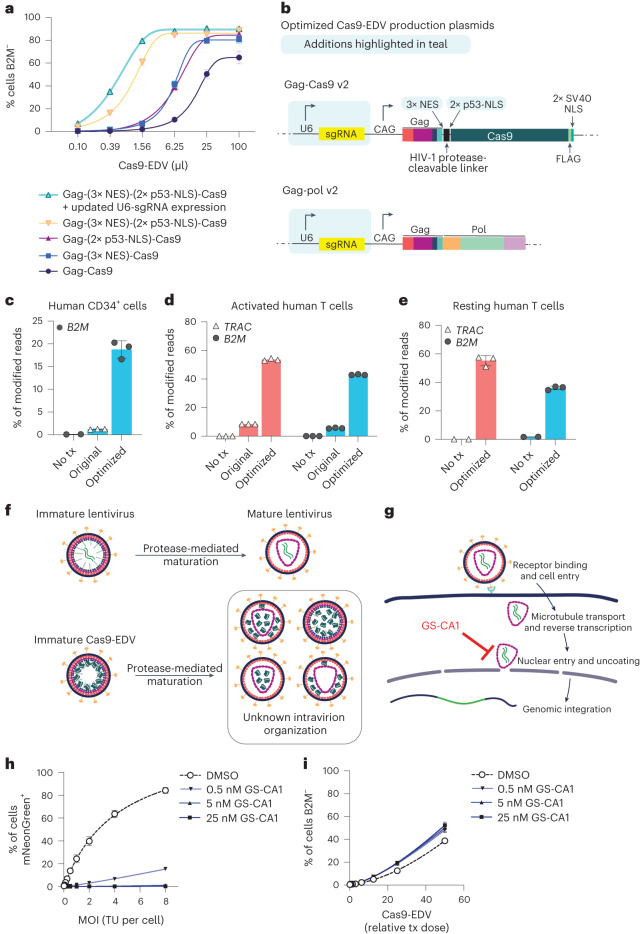
Optimization of Cas9-EDVs for enhanced genome editing activity in primary human cells. **a**, Genome editing activity comparison of CD19 antibody-targeted Cas9-EDV variants packaging *B2M-*targeted Cas9 RNPs. Expression of B2M protein was assessed by flow cytometry 7 days post treatment in CD19-expressing target cells. **b**, Diagram of the optimized Gag-Cas9 and Gag-pol Cas9-EDV production plasmids; features updated from a previous study^22^ are highlighted in teal. **c**–**e**, Genome editing activity of optimized VSVG-pseudotyped Cas9-EDVs in primary human CD34^+^ cells (**c**) and activated (**d**) and resting primary human T cells (**e**). *B2M* or *TRAC g*enome editing was assessed by amplicon sequencing 7 days post treatment; *n* = 3 technical replicates were assessed for all conditions except for the untreated resting human T cells (*n* = 2). **f**, Schematic of potential intra-particle Cas9-EDV configurations for packaged Cas9 RNPs following proteolytic maturation. **g**, Schematic of the compound GS-CA1 inhibiting either the nuclear import and/or uncoating of an HIV-1 capsid. **h**, An mNeonGreen lentiviral vector was used to transduce HEK293T cells at the indicated multiplicity of infection (MOI) in the presence of GS-CA1 or DMSO. The percent of mNeonGreen-positive cells was assessed by flow cytometry 3 days post treatment. TU, transducing units. **i**, *B2M*-targeting Cas9-EDVs, pre-titered such that the highest treatment dose would result in approximately 50% of cells B2M^−^, were used to transduce HEK293T cells in the presence of GS-CA1 or DMSO. B2M expression was assessed by flow cytometry 3 days post treatment. Error bars, s.e.m. Unless otherwise noted, *n* = 3 technical replicates were used in all experiments; four-parameter non-linear regression curves are plotted in **a**, **h** and **i**.

To enhance Cas9-EDV yield and per-particle editing efficiency ahead of in vivo administration, we screened multiple N-terminal nuclear localization signal (NLS) additions to Cas9 and found that 2× p53-derived NLS together with 3× nuclear export sequences (NESs) appended to the C-terminal end of Gag^8^ most improved the editing efficiency of antibody-targeted Cas9-EDVs (Supplementary Fig. 3a–d and Supplementary Table 2). Editing efficiency was further increased by expressing sgRNA from both the Gag-NES-NLS-Cas9 and Gag-pol plasmid backbones, as opposed to expression from a separate plasmid (Fig. 2a,b). We speculate that these optimizations improved Cas9 RNP loading into EDVs during assembly as well as enhanced Cas9 RNP nuclear import in target cells. Optimized Cas9-EDVs maintained receptor-mediated delivery specificity except at the highest doses tested (Supplementary Fig. 3e), and Cas9-EDV titration produced a 36-fold enrichment for genome editing on-target cells (79.7%) versus bystander cells (2.2%) (Supplementary Fig. 3f). Optimized Cas9-EDVs pseudotyped with the broadly transducing VSVG glycoprotein also demonstrated improved genome editing activity when tested on cytokine-stimulated primary human CD34^+^ cells and cytokine-stimulated and activated primary human T cells ex vivo (Fig. 2c,d). Surprisingly, optimized VSVG-pseudotyped Cas9-EDVs mediated genome editing in resting primary human T cells (Fig. 2e), which are difficult to edit using standard electroporation approaches. This suggests that Cas9-EDVs may be an effective strategy for genome editing T cells in the absence of cellular activation, stimulation and expansion.

We next investigated whether the internal composition of Cas9-EDVs affects editing efficiency, focusing on the lentiviral capsid that forms during proteolytic virion maturation^30^ (Fig. 2f). To probe the role of the capsid in Cas9-EDV delivery, we employed GS-CA1, a small-molecule inhibitor of nuclear import and/or subsequent uncoating of HIV-1 capsid cores^31,32^ (Fig. 2g). Treatment of target cells with increasing concentrations of GS-CA1 blocked the integration of a lentiviral transgene, which relies on nuclear import from the capsid (Fig. 2h), but did not negatively impact the genome editing efficiency of Cas9-EDVs (Fig. 2i) or electroporated Cas9 RNPs (Supplementary Fig. 3g). In a separate experiment, we generated a Cas9-EDV variant that relies on the tobacco etch virus (TEV) protease to release Cas9 from Gag (‘TEVp-Cas9-EDVs’; Supplementary Fig. 3h), preventing the HIV-1 protease-dependent virion maturation required for capsid assembly. Despite the demonstrated loss of Gag proteolytic processing (Supplementary Fig. 3i), TEVp-Cas9-EDVs maintained genome editing activity in treated cells proportional to the amount of Cas9 generated (Supplementary Fig. 3j). These results suggest that the capsid is not required for packaging and delivering Cas9 RNP complexes into target cell nuclei.

### Optimized Cas9-EDV characterization

We next performed characterization of Cas9-EDVs to better understand particle composition and genome editing activity. Cas9-EDVs are similar in diameter to lentiviral vectors (LVs) (Supplementary Fig. 4a,b), and multiple scFvs are detectable on the surface of antibody-targeted particles (Supplementary Fig. 4c). Interestingly, we could detect Cas9-independent packaging of over-expressed sgRNA into Cre recombinase-packaging Cas9-EDVs, but sgRNA packaging was enhanced ~330-fold in Cas9-containing particles (Supplementary Fig. 4d). While others have shown the unintended packaging of cellular RNAs and proteins into retroviral vectors^23,33^, which probably occurs in Cas9-EDVs, we could not detect unintended Cas9-EDV-mediated delivery of plasmids from producer cells to treated cells (Supplementary Fig. 4e,f).

Using synthetic sgRNA as a standard curve (Supplementary Fig. 4g), we estimate that Cas9-EDVs produced in one 10 cm plate contain ~2.66 × 10^11^ sgRNA molecules (Supplementary Fig. 4g) distributed among approximately 5.65 × 10^10^ Cas9-EDV particles (Supplementary Fig. 4i). Benchmarking Cas9-EDVs against Cas9 RNP nucleofection, we found that treatment with 0.2 μl of 30-fold concentrated Cas9-EDVs resulted in the equivalent amount of genome editing as ~23 pmol of nucleofected RNP (Supplementary Fig. 4j–l). Finally, we tested the biodistribution of wild-type VSVG versus VSVGmut pseudotyped LVs and found that relative to the amount of vector in the serum, both pseudotypes exhibited similar biodistribution patterns shortly after systemic administration, suggesting that VSVGmut display does not hinder in vivo particle trafficking (Supplementary Fig. 4m).

### Multiplexed targeting molecules for human T cell engineering

**Fig. 3:**
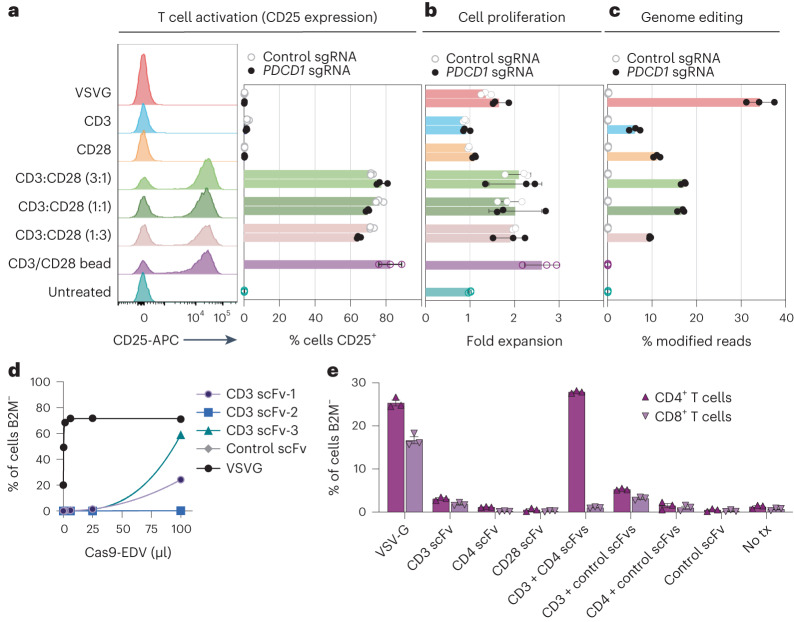
Multiplexed antibody targeting and editing of primary human T cells. **a**,**b**, Treating resting human T cells with Cas9-EDVs co-displaying CD3 and CD28 scFvs results in cellular activation (**a**) and proliferation (**b**) as measured by flow cytometry detection of CD25 3 days post treatment and fold expansion relative to the untreated T cell count, respectively. CD25 expression and cellular proliferation was observed for CD3/CD28 scFv Cas9-EDVs, regardless of whether they packaged Cas9 RNPs targeting *PDCD1* or a non-targeting control. **c**, Genome editing 3 days post treatment, as detected by amplicon next-generation sequencing. For **a**–**c**, Cas9-EDVs were concentrated 62× and 50 μl was used to treat 30,000 resting T cells. CD3 scFv-1 and CD28 scFv-2 were tested. **d**, Screening the mono-display of additional CD3 scFv targeting molecules for *B2M-*targeted Cas9-EDVs on the Jurkat T cell line. B2M expression was assessed by flow cytometry 3 days post treatment. Cas9-EDVs were concentrated 15× and 50 μl was used to treat 30,000 Jurkat cells. **e**, Testing a panel of T cell-targeted, *B2M-*targeting Cas9-EDVs, displaying single or multiplexed scFv targeting molecules. Activated primary human T cells were treated with 1.38 × 10^8^ Cas9-EDVs, displaying one or a combination of CD3 scFv-3, CD4 scFv-2, CD28 scFv-2 and a control scFv, and were assessed for B2M expression in CD4^+^ and CD8^+^ T cells by flow cytometry 6 days post treatment. Error bars, s.e.m.; *n* = 3 technical replicates were used in all experiments.

Human T cells are important targets for in vivo genome engineering applications because of their use in treating cancer and other diseases. Using CD25 expression as a marker, we found that the co-display of CD3 and CD28 targeting molecules on Cas9-EDVs triggered T cell activation and cellular expansion similar to T cells pretreated with commercially available CD3 or CD28 coated magnetic beads^34^ or engineered lentiviruses^19^ (Fig. 3a,b). CD3 + CD28 scFv Cas9-EDV treatment also led to robust levels of genome editing (Fig. 3c).

Further screening of CD3 and CD45 scFvs revealed additional Cas9-EDV targeting molecules that enabled genome editing of the human Jurkat T cell line (Fig. 3d and Supplementary Fig. 5a). The minimal human T cell editing observed with CD45-targeted Cas9-EDVs may result from a lack of CD45 internalization from the plasma membrane following monoclonal antibody engagement^35^ (Supplementary Fig. 5b,c). Primary human T cells were susceptible to genome editing using CD3-targeted Cas9-EDVs and, to a lesser extent, CD4-targeted Cas9-EDVs, but not Cas9-EDVs pseudotyped with off-target control scFv targeting molecules (Fig. 3e and Supplementary Fig. 5d). Immunophenotyping of T cells post Cas9-EDV treatment showed that CD3-targeted Cas9-EDVs direct genome editing in CD4^+^ and CD8^+^ subsets of T cells as expected, whereas CD4 scFv-targeted Cas9-EDVs specifically mediated genome editing in the CD4^+^ subset population (Fig. 3e and Supplementary Fig. 5d). Interestingly, multiplexing CD3 and CD4 scFv targeting molecules on the same Cas9-EDVs led to higher levels of editing than Cas9-EDVs displaying either CD3 or CD4 scFv targeting molecules alone (Fig. 3e and Supplementary Fig. 5d). This observation was antigen-specific, as multiplexing CD3 targeting molecules with off-target control targeting molecules did not enhance genome editing. Receptor cross-linking or aggregation can lead to endocytosis and subsequent lysosomal degradation^36,37^, possibly explaining the synergistic increase in genome editing by engaging both CD3 and CD4 receptors.

### T cell targeted Cas9-EDVs enable genome engineering in vivo

**Fig. 4:**
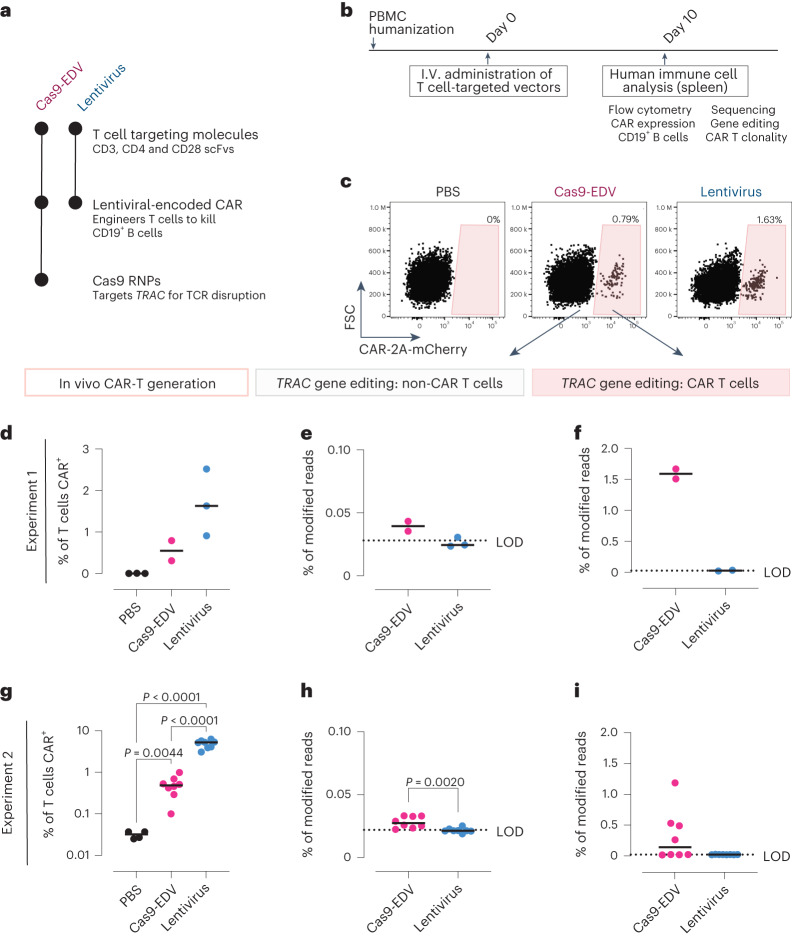
Programmable human cell delivery generates gene-edited CAR T cells *in vivo*. **a**, Summary of T cell-targeted Cas9-EDVs and lentivirus tested in PBMC-humanized mice. Both particles display multiplexed scFvs (CD3 scFv-3, CD4 scFv-1 and CD28 scFv-2). The Cas9-EDV vector co-packages a lentiviral-encoded CAR-2A-mCherry transgene and Cas9 RNP complexes to disrupt the *TRAC* gene; the lentivirus encodes the CAR-2A-mCherry transgene. **b**, Experimental schematic for testing T cell-targeted Cas9-EDVs and lentivirus in PBMC-humanized mice by intravenous (I.V.) retro-orbital injections. **c**, Representative flow cytometry plots demonstrating that CAR-expressing human T cells are detectable in the spleens of PBMC-humanized mice 10 days post administration of 1.5 × 10^9^ Cas9-EDV (*n* = 2 animals) or lentivirus (*n* = 3 animals) but not in mice administered PBS (*n* = 3), quantified in **d**. **e,f**, Gene editing is observed in CAR^−^ and CAR^+^ human T cells isolated from mice treated with T cell-targeted Cas9-EDVs (*n* = 2 animals) and T cell-targeted lentivirus (*n* = 3 animals). One CAR^+^ lentivirus sample was excluded in **f** because of failing sequencing. **g**, CAR-expressing human T cells are detectable in the spleens of PBMC-humanized mice 10 days post administration of 6.2 × 10^8^ Cas9-EDV (*n* = 8 animals) or lentivirus (*n* = 8 animals) but not in mice administered PBS (*n* = 4 animals). *P* values calculated by means of Dunnett’s multiple comparison test after Brown–Forsythe and Welsh one-way ANOVA. **h,i**, Genome editing is observed in CAR^−^ and CAR^+^ human T cells isolated from mice treated with T cell-targeted Cas9-EDVs (*n* = 8 animals per group). Significance calculated by two-sided unpaired *t*-test. Comparison in **i** is not significant (*P* > 0.05). For all plots, black lines indicate the median of the data set. LOD, limit of detection as defined by the average modified reads from lentiviral-treated samples.

Antibody-targeted EDVs have the unique flexibility of enabling cell-specific delivery of either genome editors alone, lentiviral-encoded transgenes alone or genome editors and transgenes together. We next investigated the ability of antibody-targeted EDVs to perform cell-targeted engineering of human CAR T cells in vivo, an advance that could negate the delays and costs associated with ex vivo CAR T manufacturing^38,39^. Using immunodeficient mice engrafted with human peripheral blood mononuclear cells (PBMCs) to mimic a humanized immune system, we tested T cell-targeted vectors for their ability to generate either CAR T cells (LV) or gene-edited CAR T cells (Cas9-EDV vector) in vivo (Fig. 4a). Both vectors package a lentiviral-encoded α-CD19-4-1BBz CAR-P2A-mCherry transgene, with the Cas9-EDVs additionally co-delivering Cas9 RNP complexes to disrupt the T cell receptor alpha constant (*TRAC*) gene. Both vectors rely on semi-random integration of the transgene for CAR expression and both co-display CD3, CD4 and CD28 scFvs to trigger enhanced cell entry (CD4 + CD3) as well as cell activation and proliferation (CD3 + CD28).

T cell-targeting Cas9-EDVs containing the CAR transgene (*n* = 4) or T cell-targeting lentivirus containing the CAR transgene (*n* = 3) were systemically administered and in vivo cell engineering was assessed 10 days post treatment (Fig. 4b). CAR-transduced T cells were observed in all mice in which human cells successfully engrafted, as detected by mCherry expression (Fig. 4c,d and Supplementary Fig. 6a–c). In the two Cas9-EDV-treated mice that successfully engrafted with human T cells (*n* = 2 out of 4), we observed 1.67% and 1.51% modified alleles in the CAR-transduced T cells, compared to 0.04% and 0.04% in the CAR^−^ T cells isolated from the same mice (Fig. 4e,f). As expected, no modified alleles were observed in cells isolated from mice treated with the T cell-targeted lentivirus. We repeated this experiment with mice humanized with PBMCs from a different donor and with more mice per treatment group, and we again observed CAR T cells generated in vivo in eight out of eight mice treated with T cell-targeted Cas9-EDVs and eight out of eight mice treated with T cell-targeted lentivirus (~0.5% vs ~5% CAR^+ ^ T cells, respectively) (Fig. 4g and Supplementary Fig. 6d–f). Again, we observed genome editing only in mice (*n* = 4 out of 8) treated with Cas9-EDVs, with higher levels of genome editing in CAR-transduced T cells than in CAR^−^ T cells (Fig. 4h, i). Treatment with the T cell-targeted Cas9-EDV and lentivirus was well tolerated, with no weight loss observed (Supplementary Fig. 6g). Although mCherry^+^ F4/80^+^ Kupffer cells/macrophages were observed, no mCherry^+^ β-catenin-expressing hepatocytes were detected in the liver (Supplementary Fig. 7a–d). Together, these results indicate that antibody-based targeting of Cas9-EDVs is a strategy that maintains cell-selective and tissue-specific delivery of transgenes and genome editors in vivo.

**Fig. 5:**
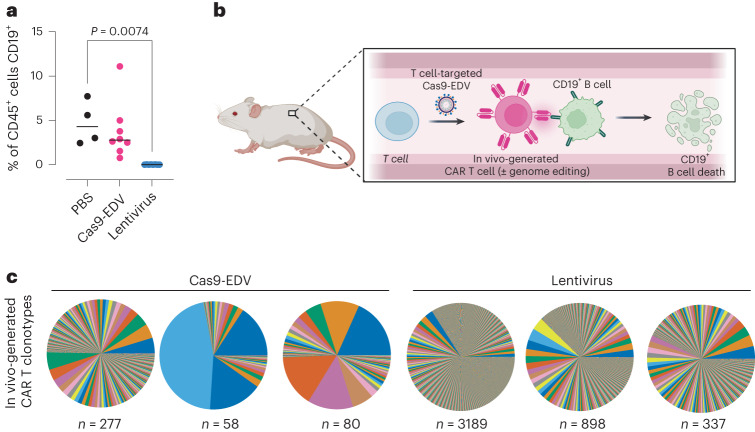
Functional dynamics of cellular engineering *in vivo*. **a**, Depletion of CD19^+^ B cells is observed post administration of T cell-targeted lentivirus (experiment 2). Human CD45^+^ cells were isolated from PBMC-humanized spleens 10 days post systemic administration of T cell-targeted Cas9-EDV (*n* = 8 animals), lentivirus (*n* = 8 animals) or PBS (*n* = 4 animals), and the percentage of CD19-expressing cells was assessed by flow cytometry. *P* values calculated by means of Dunnett’s multiple comparison test after ordinary one-way ANOVA. **P < 0.01. Black lines indicate the median of the data set. **b**, Model for the in vivo generation of CAR T cells, with or without simultaneous genome editing. Schematic made with Biorender and is not to scale. **c**, T cell receptor clonotypes of CAR-transduced T cells isolated from humanized mice treated with T cell-targeted Cas9-EDV (mouse numbers 2, 4, 6) or lentivirus (mouse numbers 13, 14, 17) from experiment 2. ‘*n*’ indicates the unique number of clonotypes detected.

The primary objective of our humanized mouse experiments was to assess Cas9-EDVs for their ability to mediate cell-targeted genome editing and transgene delivery in vivo. Given that human CD19^+^ B cells, in addition to T cells, engrafted in the second mouse cohort, we additionally assessed in vivo CAR T cell killing activity. Variable levels of CD19^+^ B cells were observed in Cas9-EDV-treated mice, and no CD19^+^ B cells were detected in mice treated with antibody-targeted lentivirus, demonstrating in vivo CAR T cell-mediated cytotoxicity (Fig. 5a and Supplementary Fig. 8a). This analysis suggests a model in which antibody-derived targeting molecules can direct molecular cargo to specific cells in vivo to successfully reprogram cell activity (Fig. 5b). Diverse T cell clonotypes were observed for CAR-transduced T cells isolated from mice in both groups (Fig. 5c), suggesting that multiple cells were engineered in vivo and did not arise solely through expansion of a single engineered cell. Given that clonotype diversity correlated with the number of CAR T cells analyzed (Supplementary Fig. 8b), the clearance of B cells in the lentiviral group was probably attributable to a higher number of CAR T cells generated during the initial in vivo transduction. Taken together, these findings offer an approach for generating genome-engineered cells with complex edits that could prove valuable for a wide range of clinical applications in the future.

## Discussion

The EDVs created here combine the molecular packaging and cellular transduction capabilities of a lentivirus with the cell-surface recognition properties of antibodies to deliver Cas9 protein, sgRNAs and transgenes into specific human cell types, both ex vivo and in vivo. Co-display of scFv antibody fragments and the VSVGmut fusogen on the Cas9-EDV envelope provides selectivity of cell transduction. Antibody-directed Cas9-EDVs mediate genome editing in targeted human cells over bystander, non-target cells in vitro and in humanized mice without transducing hepatocytes, thus avoiding a common barrier to selective in vivo delivery owing to passive liver uptake.

Cas9-EDVs enable complex cell engineering in specific cells, as shown by in vivo generation of gene-edited human CAR T cells, with important advantages relative to other in vivo delivery methods. First, unlike VLP-mediated delivery^8^, EDVs can be administered systemically for cell-type specific receptor-mediated delivery of multiple cargo molecules, including protein, RNA and DNA. Second, in contrast to viral vector-based methods for delivering DNA-encoded molecules^40–42^, EDVs provide transient delivery of preassembled genome editors whose short lifetime limits off-target editing. In addition, both adeno-associated virus and lentiviral delivery can involve random transgene integration^43,44^ that could be avoided in the future using Cas9 RNP-mediated genome editing for targeted transgene knock-in. Third, distinct from reported viral vectors^45^ and lipid nanoparticles^40^, antibody-targeted EDVs do not induce detectable transduction of liver hepatocytes, which could help avoid toxicity by minimizing the effective concentration necessary for therapeutic benefit. Finally, in contrast to previous in vivo CAR T cell generation reports using retargeted retroviruses^46,47^, there is no need for T cell activation before vector administration because Cas9-EDVs activate T cells during delivery. No treatment-related toxicity was observed following T cell-targeted vector administration in vivo; however, it will be important for future studies to assess the impact of vector dose on triggering aberrant T cell activation and proliferation.

Clonotype analysis of in-vivo-generated CAR T cells indicated that multiple independent transduction events occurred in vivo. The incomplete B cell aplasia observed for the T cell-targeted Cas9-EDV dose tested here is probably explained by the number of Cas9-EDV-generated CAR T cells being below a threshold needed for B cell ablation in the first 10 days post systemic administration. At ten days post systemic administration, we observed that 5% of all T cells were CAR^+^ in mice that were administered the T cell-targeted lentivirus compared to 0.5% in mice administered the T cell-targeted Cas9-EDV. Although this does not tell us about initial in vivo transduction rates, which could be occluded because of CAR T cell expansion, it does suggest that 5% CAR T cells at 10 days correlates with B cell aplasia in this humanized mouse model. Future experiments will assess B cell ablation either at later time points post Cas9-EDV administration to allow more time for cellular expansion or test higher doses of Cas9-EDVs to generate more CAR T cells initially. To test higher doses, future work will investigate methods for boosting the yield of antibody-targeted Cas9-EDVs, as the current process is less effective than VSVG-pseudotyped Cas9-EDV production. Additionally, it is likely that the packaging of Cas9 RNPs alongside a transgene inhibits successful gene transduction by Cas9-EDVs. Future work will also focus on optimizing the co-delivery of Cas9 RNP complexes and transgenes to improve the efficiency of Cas9-EDV gene delivery in vivo.

Two aspects of Cas9-EDV composition and cell targeting efficiency were unexpected and warrant further analysis. First, the capsid is not needed and potentially inhibits Cas9-mediated genome editing, showing that EDV-delivered Cas9 RNP complexes with nuclear localization tags are sufficient to promote nuclear access. Limiting viral structural components to those essential for EDV production may improve the per-particle delivery efficiency of Cas9-EDVs. Second, the finding that not all scFv-based targeting molecules result in equivalent levels of genome editing in target cells suggests that productive EDV delivery requires more than antigen binding. For example, CD45 does not undergo internalization upon antibody binding, which may explain the minimal editing achieved by CD45-scFv Cas9-EDVs (refs. ^35,48^). In addition, differences in scFv delivery may result from suboptimal targeting molecule display on Cas9-EDVs.

The results reported here merge the single-treatment potential of genome engineering with cell-specific delivery of preassembled genome editors to provide an approach to selective cell editing ex vivo and in vivo. Although this report focuses on the engineering of human immune cells (T cells), future work will be extended to non-immune cells, with a particular focus on the targeted engineering of tissue-resident stem cells in vivo. These findings also shed light on fundamental aspects of fusion-based cargo delivery that may be further uncovered through the investigation of EDV-based molecular trafficking, offering the potential to use EDVs for fundamental research as well as therapeutic delivery applications.

## Methods

### Plasmid construction

VSVGmut (K47A R354A VSVG) sequence was human codon-optimized and synthesized as a gBlock (Integrated DNA Technologies, IDT) and then cloned into the pCAGGS expression plasmid. To generate the CD19 scFv-1 expression plasmid, the sequence encoding the CD8*a* signal peptide, myc epitope tag, scFv and CD8*a* stalk and transmembrane domain of *a*-CD19-4-1BB**ζ**-P2A-mCherry^22,49,50^ was subcloned into pCAGGS. This plasmid was subsequently used as an entry plasmid for cloning all other scFv antibody fragments; the CD8*a* signal peptide, myc tag and scFv sequences were dropped out by EcoRI/Esp3I restriction digest (New England Biolabs, NEB) and new DNA sequences encoding CD8*a* signal peptide and scFv were inserted. This cloning strategy resulted in removing the N-terminal myc epitope tag and adding a serine amino acid residue between the scFv and CD8*a* hinge domains. A flexible linker (GGGGSGGGGSGGGGSS) was used to link VH and VL domains of source monoclonal antibody sequences. If the antibody source sequence was already an scFv, then the linker from the source sequence was used. Except for CD19 scFv-1, all antibody fragment sequences were human codon-optimized and synthesized as eBlock Gene Fragments (IDT). Lastly, a CD19 scFv expression plasmid with 2× strep-tag was generated by removing the myc tag from CD19 scFv-1 and inserting the 2× strep-tag. InFusion cloning (Takara Bio) was used to generate all plasmids. Additional information on the scFv targeting molecules and sequence sources can be found in Supplementary Table 1.

A second-generation lentiviral transfer plasmid encoding expression of EF1*a* promoter, CAR-P2A-mCherry^22^, was digested with XbaI and MluI (NEB) to drop out the CAR-P2A-mCherry transgene. Human CD19 (Uniprot no. Q71UW0) DNA was ordered as a gBlock (IDT) and IRES-EGFP (amplified from the Xlone TRE3G MCS-TEV-Halo-3XF IRES EGFP-Nuc-Puro plasmid, a gift from the Darzacq/Tijan Lab) sequences were inserted using InFusion cloning (Takara Bio). This cloning strategy inserted a MluI restriction digest site 3′ of the CD19 stop codon and removed the MluI restriction digest site 3′ of the EGFP stop codon. Human CD4 (Uniprot no. P01730), CD20 (Uniprot no. P11836) and CD28 (Uniprot no. P10747) amino acid sequences were human codon-optimized for synthesis and ordered as an eBlock (CD28) or gBlocks (CD20, CD4) (IDT). Ligand-encoding sequences were cloned by restriction digest removal of CD19-encoding sequence from the EF1*a*-CD19 IRES-EGFP lentiviral plasmid using XbaI and MluI (NEB), and inserted with InFusion cloning (Takara Bio). VSVGmut and scFv targeting plasmids were prepared using the HiSpeed Plasmid Maxi or Plasmid Plus Midi kits (Qiagen). Lentiviral plasmids were prepared with the QIAprep Spin Miniprep Kit (Qiagen). All plasmids were sequence-confirmed (UC Berkeley DNA Sequencing Facility, Quintara Bio or Primordium Labs) before use.

All Gag-fusion constructs were cloned using InFusion cloning (Takara Bio). The p53-NLS amino acid 305–322 sequence was obtained by reverse transcription PCR (RT–PCR) using RNA extracted from Raji cells as a template with the SuperScript III One-Step RT–PCR System with Platinum *Taq* DNA Polymerase (Thermo Fisher). The 2× p53-NLS was constructed by linking two p53 NLS sequences by a flexible linker (GGSGG); the 2× p53-NLS sequence was then inserted into Gag-Cas9 (Addgene plasmid no. 171060) with InFusion cloning (Takara Bio). Gag-Cas9 and Gag-(2× p53-NLS)-Cas9 were digested with MfeI-HF and AgeI-HF (NEB). The 3× NES sequence was human codon-optimized, synthesized as a gBlock (IDT) and inserted with InFusion cloning (Takara Bio) to generate Gag-(3× NES)-(2× p53-NLS)-Cas9.

The U6-sgRNA expression cassette was cloned into the plasmid backbones of Gag-(3× NES)-(2× p53-NLS)-Cas9 and psPax2 as follows: Gag-(3× NES)-(2× p53-NLS)-Cas9 was digested with SalI-HF (NEB). The Gag-pol expression plasmid psPax2 (Addgene plasmid no. 12260) was first digested with AflII and SacI (NEB) to remove a SalI restriction site. The modified psPax2 was then digested with SalI-HF (NEB). The U6-sgRNA expression cassette was amplified from the spyCas9 sgRNA-BsmBI-Destination plasmid (Addgene plasmid no. 171625) and inserted into the digested Gag-(3× NES)-(2× p53-NLS)-Cas9 and psPax2 with InFusion cloning (Takara Bio). Oligos encoding sgRNA spacers (*B2M*, 5′-GAGTAGCGCGAGCACAGCTA; *TRAC*, 5′-AGAGTCTCTCAGCTGGTACA; *PDCD1*, 5′-CGACTGGCCAGGGCGCCTGT; *tdTomato*, 5′-AAGTAAAACCTCTACAAATG; control, 5′-GTATTACTGATATTGGTGGG) were ordered from IDT, phosphorylated, annealed and ligated into BsmBI-digested sgRNA expression vectors.

The U6-B2M Gag-(3× NES)-(2× p53-NLS)-Cre plasmid was generated as follows: Gag-Cre was digested with MfeI-HF and NheI. The (3× NES)-(2× p53-NLS)-Cre sequence was synthesized as a gBlock (IDT) and inserted downstream of Gag with InFusion cloning (Takara Bio). Then, the Gag-(3× NES)-(2× p53-NLS)-Cre plasmid was digested with XbaI and PvuI-HF (NEB). The U6-B2M expression cassette was isolated by digesting U6-B2M Gag-(3× NES)-(2× p53-NLS)-Cas9 with XbaI and PvuI-HF (NEB) and inserted into the digested Gag-(3× NES)-(2× p53-NLS)-Cre with T4 DNA ligase (NEB).

Sequences for EDV production plasmids are in Supplementary Table 2.

### Tissue culture and cell line generation

Lenti-X and HEK293T cells, obtained and authenticated by the UC Berkeley Cell Culture Facility, were cultured in DMEM (Corning) supplemented with 10% fetal bovine serum (VWR) and 100 U ml^−1^ penicillin-streptomycin (Gibco) (cDMEM). To generate lentiviruses encoding EF1*a*-ligand IRES-EGFP, 3.5–4 million Lenti-X cells were plated in a 10 cm tissue culture dish (Corning) and transfected with 1 µg pCMV-VSV-G (Addgene plasmid no. 8454), 10 µg psPax2 (Addgene plasmid no. 12260), and 10 µg of EF1*a*-ligand IRES-EGFP lentiviral transfer plasmid using polyethylenimine (Polysciences) at a 3:1 PEI:plasmid ratio. Lentiviral-containing supernatants were collected 2 days post transfection and passed through a 0.45 µm PES syringe filter (VWR). Ligand-expressing cells were generated by transducing HEK293T cells (100,000 per well in a 12-well dish) with lentivirus (0.15–1 ml) in a total well volume of 1 ml. Four days post transduction, flow cytometry was used to identify cell mixtures in which <25% of cells were expressing EGFP. Following expansion, CD19 EGFP HEK293T cells were additionally sorted for EGFP expression using an SH800S cell sorter (Sony Biotechnology) to generate a population of ~100% CD19^+^EGFP^+^ cells.

### T cell culture

Cryopreserved PBMCs (AllCells) were thawed in X-VIVO 15 (Lonza) with 50 µM 2-mercaptoethanol (Gibco), 5% fetal bovine serum (VWR) and 10 mM *N*-acetyl l-cysteine (Sigma-Aldrich). This media was also used to culture the T cells isolated from PBMCs. To isolate T cells, PBMCs were incubated in 100 µg ml^−1^ DNase I solution (StemCell Technologies) at room temperature (22 °C) for 15 min and resuspended in EasySep Buffer (StemCell Technologies). Aggregated suspensions were filtered through a 37 µm cell strainer (StemCell Technologies) and incubated with EasySep Human T Cell Isolation Cocktail (StemCell Technologies). Then, EasySep Dextran RapidSpheres (StemCell Technologies) were used to separate T cells via an EasySep Magnet (StemCell Technologies). Dynabeads Human T-Activator CD3/CD28 (Gibco) and recombinant human 500 U ml^−1^ IL-2 (Peprotech), 5 ng ml^−1^ IL-7 (Peprotech) and 5 ng ml^−1^ IL-15 (R&D Systems) were used to stimulate and activate T cells for 2 days before treatment. Pre-activated T cells were cultured in media containing 500 U ml^−1^ IL-2 (Peprotech).

### CD34^+^ cell culture

Cryopreserved G-CSF-mobilized human CD34^+^ cells were acquired from AllCells. Cells were thawed, resuspended in IMDM (Gibco), spun and then cultured in StemSpan SFEM II media (StemCell Technologies) with 100 U ml^−1^ penicillin-streptomycin (Gibco) and the cytokine cocktail StemSpan CC110 (StemCell Technologies). Cells were treated with 8 µM cyclosporine H (Sigma-Aldrich) for 24 h before treatment. Additionally, cells were treated with 1 µg ml^−1^ poloxamer (BASF) at the time of treatment. Cas9-EDVs were concentrated 50-fold using Lenti-X Concentrator (Takara Bio), resuspended in SFEM II media and mixed with 40,000 CD34^+^ cells in a final well volume of 100 µl. Transduction was performed in a U-bottom 96-well plate for 24 h on an orbital shaker before cells were spun, expanded 1:1 and cultured stationary until analysis.

### Cas9-EDV production

Cas9-EDVs (formerly known as ‘Cas9-VLPs’) were produced as previously described^22^. In brief, VSVG-pseudotyped Cas9-EDVs were produced by seeding 3.5–4 million Lenti-X cells (Takara Bio) into 10 cm tissue culture dishes (Corning) and transfecting the next day with 1 µg pCMV-VSV-G (Addgene plasmid no. 8454), 6.7 µg Gag-Cas9 (Addgene plasmid no. 171060), 3.3 µg psPax2 (Addgene plasmid no. 12260) and 10 µg U6-sgRNA (Addgene plasmid no. 171635 or Addgene plasmid no. 171634) using polyethylenimine (Polysciences) at a 3:1 PEI:plasmid ratio. Antibody-targeted Cas9-EDVs were produced in the same way as VSVG Cas9-EDVs, except that the pCMV-VSV-G plasmid was omitted and 7.5 µg of scFv targeting plasmid and 2.5 µg of VSVGmut were included during transfection. For scFv multiplexing, a total of 7.5 µg scFv plasmids was used, split 1:1 or 1:1:1 unless otherwise described in the figure legend. For Cas9-EDVs used to treat primary human cells (T cells, CD34^+^ cells) or humanized mice, media was changed 6–18 h post transfection into Opti-MEM (Gibco). Two days post transfection (or media change), Cas9-EDV-containing supernatants were collected, passed through a 0.45 µm PES syringe filter (VWR) and concentrated with Lenti-X Concentrator (Takara Bio) according to the manufacturer’s instructions. Concentrated Cas9-EDVs were resuspended in Opti-MEM (Gibco) at a final concentration of 10× unless otherwise noted in the figure legend. Cas9-EDVs were stored at 4 °C or frozen at −80 °C within an isopropanol-filled freezing container until use.

To optimize Cas9-EDVs, variants with different Gag-Cas9 polypeptides were produced in the same way as described above, except that 6.7 µg of each Gag-Cas9 variant (Gag-(3× NES)-Cas9, Gag-(2× p53-NLS)-Cas9 and Gag-(3× NES)-(2× p53-NLS)-Cas9) was used instead of Gag-Cas9 during transfection. Gag-(3× NES)-(2× p53-NLS)-Cas9 EDVs with U6-sgRNA expression from plasmid backbones were produced similarly, except that the U6-B2M plasmid was omitted, and 6.7 µg of U6-sgRNA Gag-(3× NES)-(2× p53-NLS)-Cas9 and 3.3 µg of U6-sgRNA psPax2 were included instead of the Gag-Cas9 and psPax2 plasmids during transfection. To capture both improvements in Cas9-EDV particle production and the per-particle editing efficiency of Cas9-EDVs, we produced equivalent numbers of transfected 10 cm tissue culture dishes (Corning) of each Cas9-EDV variant tested.

For humanized mouse experiments, Cas9-EDVs were produced by transfecting Lenti-X cells with 2.5 µg of each scFv targeting plasmid (CD3 scFv-3, CD4 scFv-1 and CD28 scFv-2), 2.5 µg of VSVGmut, 3.3 µg of U6-TRAC Gag-(3× NES)-(2× p53-NLS)-Cas9, 6.7 µg of U6-TRAC psPax2 and 2.5 µg of the lentiviral transfer plasmid encoding an α-CD19-4-1BBz CAR-P2A-mCherry transgene, as optimized in a previous study^22^. Lentivirus was produced in the same way, except that U6-TRAC Gag-(3× NES)-(2× p53-NLS)-Cas9 and U6-TRAC psPax2 were omitted and 10 µg psPax2 was included. Cas9-EDV-containing and LV-containing supernatants were passed through a 0.45 µm PES filter bottle (Thermo Fisher) and concentrated through ultracentrifugation by floating the supernatant on top of a cushioning buffer of 30% (w/v) sucrose in 100 mM NaCl, 10 mM Tris-HCl pH 7.5, 1 mM EDTA pH 8.0, at 85,000×*g* with a SW28 Ti rotor (Beckman Coulter) for 2 h at 4 °C in polypropylene tubes (Beckman Coulter). After ultracentrifugation, the Cas9-EDV pellet was resuspended in sterile Dulbecco’s phosphate-buffered saline (DPBS) (Gibco).

### Cas9 RNP electroporation

*B2M*-targeting crRNA (IDT) and tracrRNA (IDT, no. 1072534) were resuspended in IDT duplex buffer to 160 µM, combined at a ratio of 1:1 and annealed at 37 °C for 30 min. Cas9 RNPs were formed by combining the annealed crRNA and tracrRNA and 40 µM Cas9-NLS (UC Berkeley QB3 MacroLab) at a molar ratio of 2:1 and incubating at 37 °C for 15 min. Electroporation was performed using a 96-well format 4D-nucleofector (Lonza) with 200,000 cells per well. HEK293T cells were electroporated with the SF buffer and the CM-130 pulse code, and primary human T cells were electroporated with the P3 buffer and the EH-115 pulse code. Cells were immediately resuspended in pre-warmed media, incubated for 20 min and transferred to culture plates.

### Cas9-EDV titer quantification

The QuickTiter Lentivirus Titer Kit (Lentivirus-Associated HIV p24) (Cell Biolabs) was used to quantify Cas9-EDV particle number. Cas9-EDVs were diluted 1:1,00–100,000, and ELISA was performed according to the manufacturer’s directions. Absorbance at 450 nm was measured by a plate reader (BioTek). Cas9-EDV p24 content was calculated by comparison to serial dilution of a p24 standard, and guidance from the manufacturer was used to convert p24 quantity into particle number (Cell Biolabs).

### Western blot analysis

Cas9-EDVs were mixed with Laemmli buffer containing 10% 2-mercaptoethanol and heating at 90 °C for 5 min. Proteins from whole cell lysates were separated by 4%–20% SDS–PAGE gel (Bio-Rad) and transferred to an Immun-Blot low fluorescence PVDF membrane (Bio-Rad). Membranes were blocked and incubated with primary antibodies at 4 °C overnight followed by secondary antibodies at room temperature for 1 h. Primary and secondary antibodies are listed in Supplementary Table 3. Imaging was performed using the Odyssey imaging system (LI-COR).

### GS-CA1 experiments

To generate lentiviruses encoding EF1*a*-mNeonGreen, 3.5–4 million Lenti-X cells were plated in a 10 cm tissue culture dish (Corning) and transfected with 1 µg pCMV-VSV-G (Addgene plasmid no. 8454), 10 µg psPax2 (Addgene plasmid no. 12260) and 2.5 µg of EF1*a*-mNeonGreen lentiviral transfer plasmid using polyethylenimine (Polysciences) at a 3:1 PEI:plasmid ratio. Lentiviral-containing supernatants were collected 2 days post transfection and passed through a 0.45 µm PES syringe filter (VWR). Lentiviral supernatants were concentrated 20× with Lenti-X Concentrator (Takara Bio) according to the manufacturer’s instructions, resuspended in Opti-MEM (Gibco), aliquoted and frozen at −80 °C for future use. *B2M*-targeted VSVG Cas9-EDVs were produced as described above. The mNeonGreen lentivirus stock was pre-titered on HEK293T cells; Cas9-EDV and mNeonGreen lentivirus samples were diluted in Opti-MEM in a twofold dilution series. Aliquots of 50 µl of each dilution series were mixed with 15,000 HEK293T cells in 50 µl cDMEM in triplicate in a 96-well plate. For the lentiviral sample, the percentage of mNeonGreen^+^ cells was assessed by flow cytometry 3 days post transduction. Wells in which the percent of mNeonGreen^+^ cells was ≤25% were used to calculate the transducing units per ml. The genome editing activity of the Cas9-EDV stock was pre-titered similarly, except that B2M expression was assessed by flow cytometry at 3 days post treatment to calculate Cas9-EDV volume that resulted in approximately 50% of cells negative for B2M expression.

The HIV-1 capsid inhibitor GS-CA1 (Gilead Sciences) was diluted to a working stock of 100 µM using DMSO. A total of 15,000 HEK293T cells were transduced with mNeonGreen lentivirus or *B2M-*targeting Cas9-EDVs and simultaneously treated with 0, 0.5, 5 or 25 nM GS-CA1 in a total well volume of 100 µl (0.05% DMSO final). mNeonGreen and B2M expression were assessed by flow cytometry 3 days post treatment. As a control, *B2M* sgRNA and Cas9 protein (IDT) were complexed at a 2:1 ratio for 15 min at 37 °C, and 50 pmol Cas9 RNPs were nucleofected into 200,000 HEK293T cells using the SF Cell Line 4D-Nucleofector Kit and 4D-Nucleofector instrument (Lonza), using pulse code CM-130. Post nucleofection, cells were brought up to 100 µl with pre-warmed cDMEM and incubated at 37 °C for 15 min. Nucleofected cells were plated at 15,000 per well of a 96-well plate and treated with 25 nM GS-CA1, 0.05% DMSO or Opti-MEM. B2M expression was assessed by flow cytometry 3 days post nucleofection.

### Humanized mouse experiments

All animal studies and procedures were conducted in accordance with the established National Institutes of Health guidelines for animal care and use and were approved by the UC Berkeley Animal Care and Use Committee. All experimental and control animals were housed under the same conditions as approved by the Berkeley Office of Laboratory Animal Care. Mice were housed at ambient room temperature in a humidity-controlled animal facility with free access to water and food. Mice were maintained on a 12:12 h light:dark cycle (lights on from 07:00 to 19:00 h).

Human peripheral blood mononuclear cell-engrafted NSG mice (745557, 6–8 weeks of age) were purchased from Jackson Laboratory. ‘Experiment 1’ and ‘experiment 2’ mouse cohorts were engrafted with cells from unique human donors. Following anesthesia induction with 2–3% isofluorane, 100 µl of Cas9-EDVs, lentivirus or PBS was administered by retro-orbital injection. Mice were euthanized with CO_2_ 10 days post treatment and the spleen and liver were dissected. See ‘Immunofluorescent staining and imaging’ for downstream liver sample processing. Single-cell suspensions of spleen were prepared by gently bursting the organ in 4 ml DMEM (Corning) in a well of a 6-well dish using the back of a 3 ml syringe (Thermo Fisher). Splenocytes were passed through a 100 μm cell mesh (Corning), brought up to 25 ml with DPBS (Gibco) and spun at 300×*g* for 10 min. Erythrocytes were lysed by resuspending splenocytes in 5 ml 1× BD Pharm Lyse lysing solution (BD Biosciences) for 5 min before being brought up to 25 ml with DPBS. Cells were then pelleted at 300×*g* for 10 min, resuspended in 10 ml DPBS and counted using a Countess 3 automated cell counter (Thermo Fisher). Cells were cryopreserved in freeze media (Bambanker) for downstream cell sorting. For flow cytometry analysis, CD45^+^ cells were isolated from splenocytes using the EasySep Release Human CD45 Positive Selection Kit for Humanized Mouse Samples (StemCell Technologies) on an EasySep EasyEights Magnet (StemCell Technologies) according to the manufacturer’s instructions. Immunophenotyping was performed using anti-human CD4-FITC, anti-human CD4-PE-Cyanine7, anti-human CD8-PE-Cyanine7 and anti-human CD19-FITC (Supplementary Table 3), and cells were assessed for CAR-2A-mCherry expression using an Attune NxT flow cytometer with 96-well autosampler (Thermo Fisher). For cell sorting, cryopreserved splenocytes were thawed and stained with anti-human CD4-FITC and anti-human CD8-FITC (Supplementary Table 3) in PBS containing 1% BSA, and an SH800S cell sorter (Sony Biotechnology) was used to sort mCherry^+^ and mCherry^−^ primary human T cells that were either expressing CD4 or CD8.

### Flow cytometry

Cells were stained with anti-human B2M-APC or anti-human B2M-PE (Supplementary Table 3) in PBS containing 1% BSA, and an Attune NxT flow cytometer with 96-well autosampler (Thermo Fisher) was used for flow cytometry analysis. Ligand expression was confirmed for engineered HEK293T cells using anti-human CD28-PE, anti-human CD20-PE, anti-human CD4-PE-Cyanine7 and anti-human CD19-PE (Supplementary Table 3). T cell immunophenotyping was performed using anti-human CD3-FITC, anti-human CD4-FITC and anti-human CD8-PE-Cyanine7 (Supplementary Table 3). CD25 expression was assessed using anti-human CD25-APC (Supplementary Table 3). Data analysis was performed using FlowJo v.10.7.1 (FlowJo).

### Amplicon sequencing to assess genome editing

Next-generation sequencing was used to detect on-target genome editing in EGFP^+^ and EGFP^−^ sorted HEK293T cells. Genomic DNA was extracted using QuickExtract (Lucigen) as previously described^22^. PrimeStar GXL DNA polymerase (Takara Bio) was used to amplify the Cas9 RNP target site using the following primers: *B2M*, 5′-GCTCTTCCGATCTaagctgacagcattcgggc and 5′-GCTCTTCCGATCTgaagtcacggagcgagagag; *PDCD1*, 5′-GCTCTTCCGATCTccgaccccacctacctaaga and 5′-GCTCTTCCGATCTgacagtttcccttccgctca. The resulting PCR products were cleaned up using magnetic SPRI beds (UC Berkeley DNA Sequencing Facility). The Innovative Genomics Institute Next-Generation Sequencing Core performed library preparation and sequencing using a MiSeq v2 Micro 2×150bp kit (Illumina). Reads were trimmed and merged (Geneious Prime, v.2022.0.1) and analyzed with CRISPResso2 (http://crispresso.pinellolab.partners.org/login).

### NextSeq P2 sequencing of sorted mouse cells

Genomic DNA was extracted from sorted mCherry^+^ and mCherry^−^ primary human T cells using the QIAamp DNA Mini Kit (Qiagen) according to the manufacturer’s instructions. PrimeStar GXL DNA polymerase (Takara Bio) was used to amplify the *TRAC* Cas9 RNP target site using primers 5′-GCTCTTCCGATCTggggcaaagagggaaatgaga and 5′-GCTCTTCCGATCTactttgtgacacatttgtttgag. The resulting PCR products were cleaned up using magnetic SPRI beds (UC Berkeley DNA Sequencing Facility). The Innovative Genomics Institute Next-Generation Sequencing Core performed library preparation and sequencing using a NextSeq 1000/2000 P2 v3 2×150bp kit (Illumina). Reads were trimmed and merged (Geneious Prime, v.2022.0.1) and analyzed with CRISPResso2 (http://crispresso.pinellolab.partners.org/login).

### TCR sequencing

Splenocytes from three Cas9-EDV-treated mice and three lentivirus-treated mice in humanized mouse experiment 2 were sorted into mCherry^+^ and mCherry^−^ primary human T cells using an SH800S cell sorter (Sony Biotechnology). RNA was extracted from sorted cells using the RNeasy Mini Kit (Qiagen) according to the manufacturer’s instructions. TCR a/b libraries were prepared from each sample using the SMART-Seq Human TCR (with UMIs) kit (Takara Bio) according to the manufacturer’s instructions. Samples were pooled and sequenced on an Illumina MiSeq using 2×300 bp paired-end chemistry and MiSeq Reagent Kit v.3 (MS-102-3003, Illumina), yielding an average sequencing depth of 1.75 million reads. FASTQ files were analyzed with Cogent NGS Immune Profiler Software (v.1.5), using a UMI cutoff of 3. Clonotypes were visualized using Cogent NGS Immune Viewer (v.1.0) and a custom Python script.

### Immunofluorescent staining and imaging

Humanized mice were killed with CO_2_ and livers were dissected. Livers were fixed in 4% paraformaldehyde (Electron Microscopy Sciences) for 24 h and transferred to 30% sucrose (Fisher Chemical). After 3 days, livers were embedded into cryoblocks using the Tissue Plus O.C.T. Compound (Fisher HealthCare). Liver sections (20 µm) were cut with the Leica CM 3050 S Cryostat, placed on Superfrost Plus microscope slides (Fisher Scientific) and stored at −80 °C.

Sections were blocked with a buffer including 5% normal goat serum (Sigma-Aldrich), 2% BSA (Sigma-Aldrich), 0.03% Triton X-100 (Fisher Scientific) and 0.05% sodium azide (Sigma-Aldrich) for 1 h at room temperature. Sections were stained for 2 h at room temperature with primary antibodies as follows: mouse anti-β-catenin and rat anti-F4/80; then mouse anti-β-catenin and rabbit anti-hCD3 (Supplementary Table 3). Tissue sections were washed three times with 1× PBS and stained with secondary antibodies for 1 h (Supplementary Table 3). Tissue sections were washed again three times with 1× PBS and treated with DAPI (Sigma-Aldrich) for 10 min. Then, sections were covered with cover glass slip (Micro Cover Glass, VWR) with Fluoromount-G (Southern Biotech). For the negative control, the sections were treated with secondary antibodies only and DAPI (Sigma-Aldrich).

The images of stained liver sections were taken at ×20 magnification with the Echo Revolve fluorescent microscope and obtained using the associated software (ECHOPro) through DAPI, FITC, Texas Red and CY5 channels.

### Immunogold staining

Cas9-EDVs with CD19 scFvs with either strep or myc epitope tags were produced as stated above. Aliquots of 25 ml of the Cas9-EDVs were concentrated using ultracentrifugation at 100,000×*g* for 75 min through 9 ml of a 10% v/v iodixanol cushion (StemCell Technologies) in 1× PBS. The supernatant was removed and the Cas9-EDVs were resuspended in 100 µl of 10 mM Tris-HCl pH 7.5, 150 mM NaCl. Cas9-EDVs were stored at 4 °C and used within 48 h. Carbon Type-B copper transmission electron microscopy grids (Ted Pella) were glow-discharged. Then, 5 µl of the EDVs were applied to the carbon side of the grid and incubated for 3 min. Excess sample was removed by blotting with Kimwipes. The grids were blocked for 2 min using 15 µl of 10 mM Tris-HCl pH 7.5, 150 mM NaCl and 1% w/v BSA (Sigma-Aldrich) (blocking buffer). The excess blocking buffer was removed by blotting with Kimwipes. Anti-myc antibody (15 µl of 50 µg ml^−1^; Supplementary Table 3) was applied to the grid and incubated for 30 min at room temperature. A lid was used to cover the grids and prevent them from drying out. The grids were subsequently washed five times with 15 µl of blocking buffer. Then 12 µl of 1/10 diluted goat anti-mouse 6-nm immunogold conjugates (Electron Microscopy Sciences) was applied to the grids and incubated for 30 min at room temperature. The grids were subsequently washed with 15 µl of blocking buffer four times and with ultrapure water once. Uranyl formate (0.7%, 5 µl; Ted Pella) was applied to stain and fix the samples. Excess stain was removed by blotting with Kimwipes. The EDVs were visualized using a FEI Tecnai T12 transmission electron microscope operating at 120 kV and a Gatan UltraScan 895 4k CCD (UC San Francisco EM Core).

### Dynamic light scattering

Cas9-EDVs and LVs were assessed using a Zetasizer Nano ZS (Malvern Panalytical) instrument with plastic micro cuvettes (Malvern Panalytical). Cas9-EDVs and LVs were produced as described above, except that 6–18 h post transfection of Lenti-X cells, media was changed into 5 ml Opti-MEM instead of 10 ml per 10 cm tissue culture dish. Two days post media change, Cas9-EDV-containing or LV-containing supernatants were collected and passed through a 0.45 µm PES syringe filter (VWR) without further concentration. EDV and LV particle numbers were measured and normalized using the QuickTiter Lentivirus Titer Kit (Lentivirus-Associated HIV p24) as described above. For dynamic light scattering measurements, 40 μl of normalized Cas9-EDV and LV were prepared, and particle size was measured at 25 °C. As a control, 100 nm diameter NanoXact Gold Nanospheres – Bare (Citrate) (nanoComposix) were included. Data were analyzed by intensity using the Zetasizer analysis software.

### Quantitative RT–PCR of *B2M* sgRNA

Cas9-EDVs containing gRNA targeting the *B2M* gene were produced and concentrated as described above, and RNA was extracted from 150 μl of Cas9-EDVs using the NucleoSpin RNA Virus Kit (Takara Bio) according to the manufacturer’s instructions. Quantitative RT–PCR (RT–qPCR) was performed using the PrimeTimeTM One-Step RT–qPCR Master Mix (IDT) according to the manufacturer’s instructions on a QuantStudio 6 Flex Real-Time PCR System (Thermo Fisher). The qPCR primers were ordered as a custom TaqMan Small RNA Assay to detect the *B2M* sgRNA target sequence GAGUAGCGCGAGCACAGCUAGUUUAAGAGCUAUGCUGGAAACAGCAUAGCAAGUUUAAAUAAGGCUAGUCCGUUAUCAACUUGAAAAAGUGGCACCGAGUCGGUGC (Thermo Fisher, Assay ID CTWCW3V).

### Biodistribution experiment

A total of 100 µl of lentivirus displaying either VSVG or CD19^+^VSVGmut, or PBS was administered to C57BL/6 mice (000664, Jackson Laboratory, 9–10 weeks of age) by retro-orbital injection following anesthesia induction with 2–3% isofluorane. Mice were killed with CO_2_ 30 min post treatment and spleen, kidney, heart, liver, lung and blood were collected. Spleen, kidney, heart, liver and lung samples (15 mg each) were collected and stored in 1× DNA/RNA Shield (Zymo Research). Blood samples were drawn into tubes pre-coated with 0.5 M EDTA and were then separated into plasma and red blood cells by carefully layering blood diluted with PBS containing 2% FBS on top of Percoll density gradient media (Cytiva) and centrifuging at 800×*g* for 20 min. Plasma and red blood cell layers were extracted and mixed with an equal volume of 2× DNA/RNA Shield (Zymo Research). RNA was extracted from tissue samples using the Quick-RNA Viral Kit (Zymo Research) according to the manufacturer’s instructions. Lentivirus titers were measured using the Lenti-X qRT–PCR Titration Kit (Takara Bio) on a QuantStudio 3 Real-Time PCR System (Thermo Fisher).

### Statistical analysis

Statistical analysis was performed using Prism v.9. Statistical details for all experiments, including the value and definition of sample sizes and error bars, can be found in the figure legends.

### Reporting summary

Further information on research design is available in the Nature Portfolio Reporting Summary linked to this article.

## Online content

Any methods, additional references, Nature Portfolio reporting summaries, source data, extended data, supplementary information, acknowledgements, peer review information; details of author contributions and competing interests; and statements of data and code availability are available at 10.1038/s41587-023-02085-z.

## Supplementary information

Supplementary Information Supplementary Figs. 1–8.

Reporting Summary

Supplementary Table 1 Information on single-chain variable fragment targeting molecule sequences.

Supplementary Table 2 Transgene sequences for Cas9-EDV production.

Supplementary Table 3 Information on flow cytometry antibodies used in this study.

Supplementary Data 1 Statistical source data for supplementary figures.

## Source data

Source Data Fig. 1 Statistical source data for main figures.

## Data Availability

Sequencing data have been deposited in the National Institutes of Health NCBI SRA (BioProject PRJNA1023251) and GEO (accession number GSE235643) repositories^51,52^. Flow cytometry raw data files are available upon request. All other data are available in the main text or the Supplementary Information. Plasmids generated in this study are available on Addgene.

## Code availability

Code generated for this work has been deposited online and is publicly available on Github (10.5281/zenodo.8417852)^53^.
