# Supplementary material for: In vivo human T cell engineering with enveloped delivery vehicles
Source: Nat Biotechnol. 2024 Jan 11;42(11):1684–92. doi: 10.1038/s41587-023-02085-z (PMC11236958; doi:10.1038/s41587-023-02085-z)
Supplement: Supplementary file 1 — Supplementary Figs. 1–8. [file 41587_2023_2085_MOESM1_ESM.pdf]

nature biotechnology

Article https://doi.org/10.1038/s41587-023-02085-z

In vivo human T cell engineering with enveloped delivery vehicles

In the format provided by the authors and unedited

Table of Contents

	Page
**Supplementary Fig. 1.** Establishing antibody-targeted Cas9-EDV delivery.	2
**Supplementary Fig. 2.** Antibody-targeted Cas9-EDVs are a programmable strategy for mediating genome editing in specific cells.	3-4
**Supplementary Fig. 3.** Optimization and study of Cas9-EDV activity.	5-6
**Supplementary Fig. 4.** Optimized Cas9-EDV characterization and *in vivo* biodistribution.	7-8
**Supplementary Fig. 5.** Assessment of T cell-targeting molecules for Cas9-EDV delivery.	9
**Supplementary Fig. 6.** Assessment of CAR T generation *in vivo* using T cell-targeted vectors.	10-11
**Supplementary Fig. 7.** Analysis of delivery to off-target cells *in vivo.*	12-13
**Supplementary Fig. 8.** Assessment of CD19+ B cells and CAR T cell receptor clonality following *in vivo* cellular engineering.	14
**Supplementary Information References**	15

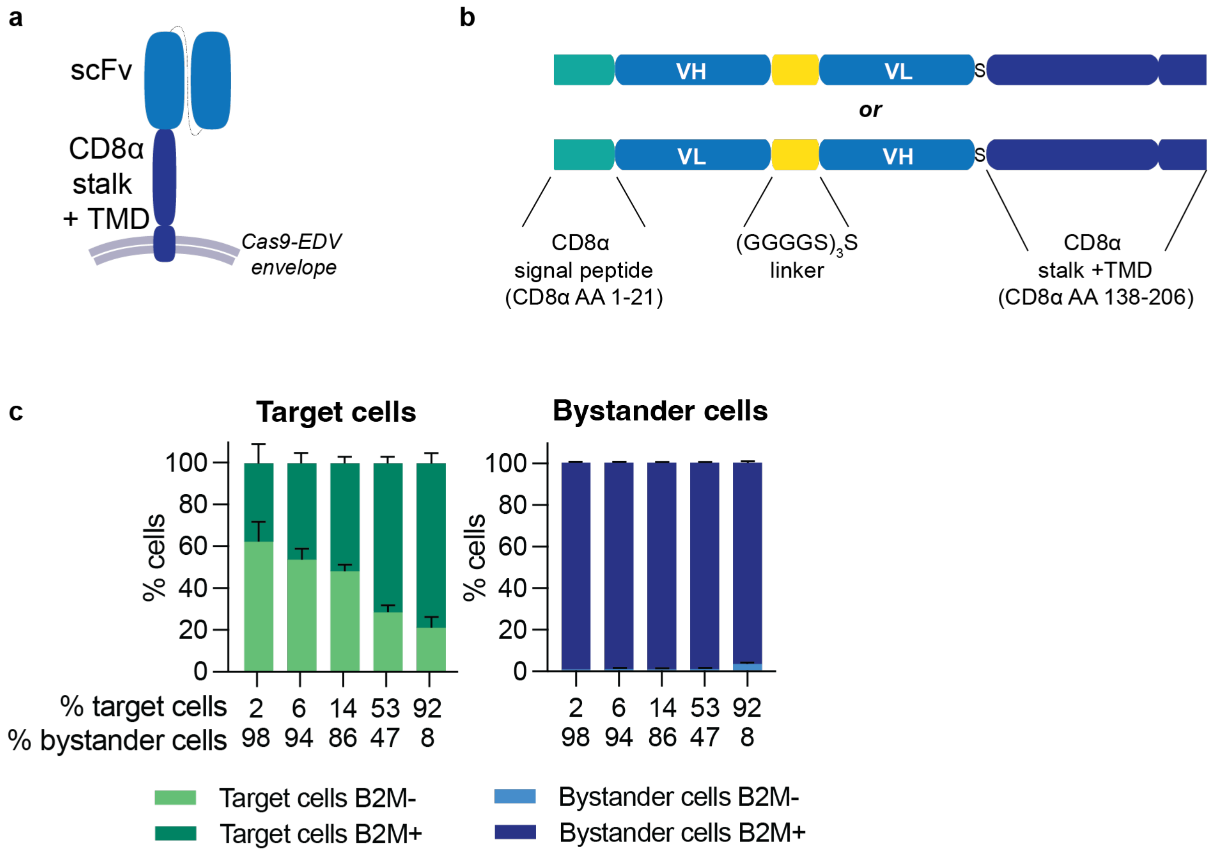

Supplementary Fig. 1. Establishing antibody-targeted Cas9-EDV delivery. a, Schematic of an antibody-derived single-chain variable fragment (scFv) targeting molecule for Cas9-EDV display. The scFv is fused to the stalk and transmembrane domain (TMD) of human CD8α. **b,** Single-chain variable fragment (scFv) targeting molecule schematic. ScFv targeting molecules were constructed in either VH-VL or VL-VH orientations. Amino acid (AA) residues correspond to the CD8α protein sequence (UniProt P01732). TMD = transmembrane domain. S = serine. **c**, Antibody-targeted Cas9-EDVs mediate targeted genome editing regardless of target cell frequency. CD19 EGFP HEK293T cells were mixed with HEK293T cells to achieve target cell frequencies of ~2-92%. Heterogeneous cell mixtures were challenged with antibody-targeted Cas9-EDVs (100 µl, 2.5x concentration), and *B2M* knockout was assessed in EGFP+ (on-target) and EGFP- (bystander) cells by flow cytometry 7 days post-treatment. N=3 technical replicates. Error bars represent the standard error of the mean.

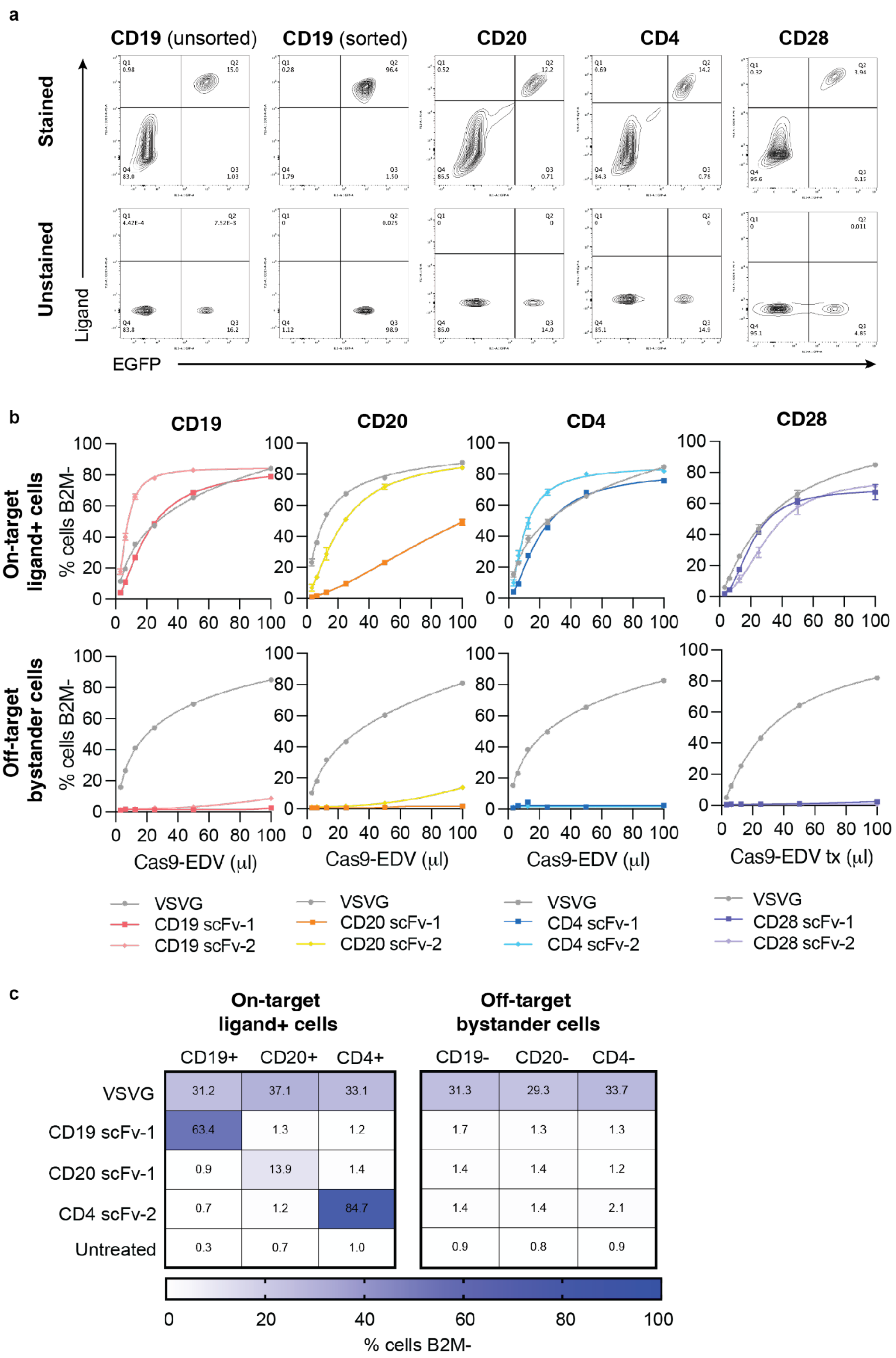
a
CD19 (unsorted)
CD19 (sorted)
CD20
CD4
CD28
Stained
Unstained
Ligand
EGFP
b
CD19
CD20
CD4
CD28
On-target ligand+ cells
Off-target bystander cells
% cells B2M-
Cas9-EDV (µl)
Cas9-EDV tx (µl)
VSVG
CD19 scFv-1
CD19 scFv-2
CD20 scFv-1
CD20 scFv-2
CD4 scFv-1
CD4 scFv-2
CD28 scFv-1
CD28 scFv-2
c
On-target ligand+ cells
Off-target bystander cells
CD19+ CD20+ CD4+
CD19- CD20- CD4-
VSVG 31.2 37.1 33.1 31.3 29.3 33.7
CD19 scFv-1 63.4 1.3 1.2 1.7 1.3 1.3
CD20 scFv-1 0.9 13.9 1.4 1.4 1.4 1.2
CD4 scFv-2 0.7 1.2 84.7 1.4 1.4 2.1
Untreated 0.3 0.7 1.0 0.9 0.8 0.9
0 20 40 60 80 100
% cells B2M-

Supplementary Fig. 2. Antibody-targeted Cas9-EDVs are a programmable strategy for mediating genome editing in specific cells. a, Validation of cell-surface ligand expression in engineered cells. Engineered 293T cells transduced to express either CD19, CD20, CD4, or CD28, and EGFP were assayed for successful ligand expression. Cells were stained with monoclonal antibodies (CD19-PE, CD20-PE CD4-PE-Cy7, CD28-PE) to verify ligand expression only in EGFP+ cells. **b,** Various scFv targeting molecules were developed to target ligands expressed on human immune cells: CD19, CD20, CD4, and CD28. Cell-specific antibody-retargeted Cas9-EDVs activity was assessed for on-target, ligand+ cells (EGFP+) and off-target, bystander cells (EGFP-) by flow cytometry 7 days post-treatment. **c,** Antibody-targeted Cas9-EDV-mediated genome editing is highly specific for cells expressing the scFv cognate ligand. scFv-Cas9-EDVs were tested on engineered cells expressing the cognate and noncognate ligands, and genome editing activity was measured by flow cytometry 3 days post-treatment. N = 3 technical replicates. All error bars represent the standard error of the mean.

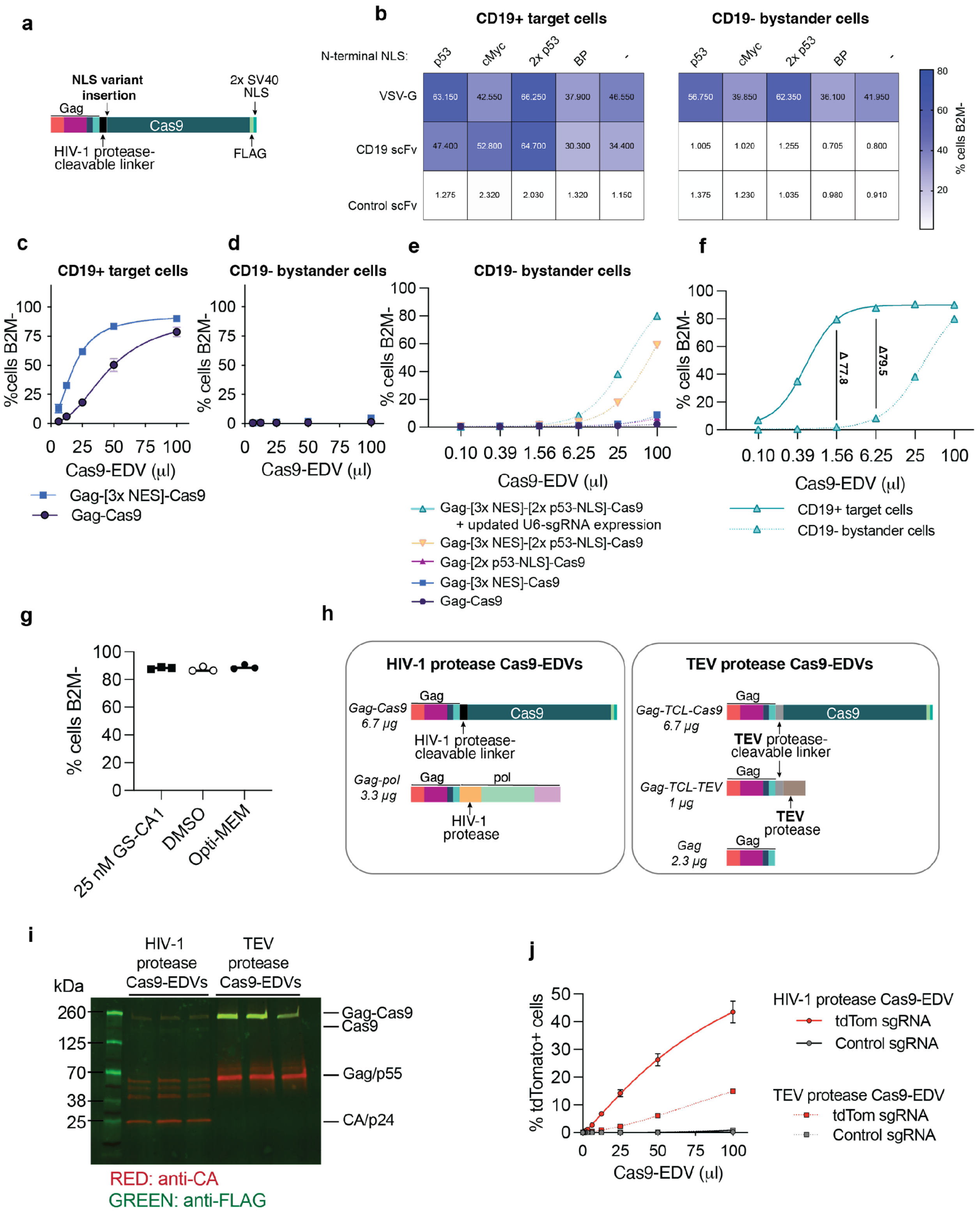
a
NLS variant insertion
2x SV40 NLS
Gag
Cas9
HIV-1 protease-cleavable linker
FLAG
b
CD19+ target cells
CD19- bystander cells
N-terminal NLS:
p53
cMyc
2x p53
BP
-
VSV-G
63.150
42.550
66.250
37.900
46.550
56.750
39.850
62.350
36.100
41.950
CD19 scFv
47.400
52.800
64.700
30.300
34.400
1.005
1.020
1.255
0.705
0.800
Control scFv
1.275
2.320
2.030
1.320
1.150
1.375
1.230
1.035
0.980
0.910
% cells B2M-
c
CD19+ target cells
%cells B2M-
Cas9-EDV (μl)
Gag-[3x NES]-Cas9
Gag-Cas9
d
CD19- bystander cells
% cells B2M-
Cas9-EDV (μl)
e
CD19- bystander cells
% cells B2M-
Cas9-EDV (μl)
Gag-[3x NES]-[2x p53-NLS]-Cas9
+ updated U6-sgRNA expression
Gag-[3x NES]-[2x p53-NLS]-Cas9
Gag-[2x p53-NLS]-Cas9
Gag-[3x NES]-Cas9
Gag-Cas9
f
% cells B2M-
Δ 77.8
Δ79.5
Cas9-EDV (μl)
CD19+ target cells
CD19- bystander cells
g
% cells B2M-
25 nM GS-CA1
DMSO
Opti-MEM
h
HIV-1 protease Cas9-EDVs
Gag-Cas9
6.7 μg
Gag
Cas9
HIV-1 protease-cleavable linker
Gag-pol
3.3 μg
pol
HIV-1 protease
TEV protease Cas9-EDVs
Gag-TCL-Cas9
6.7 μg
TEV protease-cleavable linker
Gag-TCL-TEV
1 μg
TEV protease
Gag
2.3 μg
i
HIV-1 protease Cas9-EDVs
TEV protease Cas9-EDVs
kDa
260
125
70
38
25
Gag-Cas9
Cas9
Gag/p55
CA/p24
RED: anti-CA
GREEN: anti-FLAG
j
% tdTomato+ cells
Cas9-EDV (μl)
HIV-1 protease Cas9-EDV
tdTom sgRNA
Control sgRNA
TEV protease Cas9-EDV
tdTom sgRNA
Control sgRNA

Supplementary Fig. 3. Optimization and study of Cas9-EDV activity. **a**, Diagram of the Gag-Cas9 plasmid indicating the site of nuclear localization signal (NLS) variant addition to the N-terminal end of Cas9. Sequences are listed in **Supplementary Table 2**. **b**, Assessment of N-terminal Cas9 NLS additions on Cas9-EDV activity. Cas9-EDVs were used to treat a mixture of CD19+ and CD19- 293T cells. Genome editing was assessed by flow cytometry detection of B2M expression in CD19+ target cells and CD19- bystander cells 7 days post-treatment. BP = bipartite. **c,d**, Direct comparison of genome editing in CD19+ on-target cells (**c**) and CD19- bystander cells (**d**) treated with Gag-[3x NES]-Cas9 EDVs versus Gag-Cas9 EDVs. Genome editing was assessed by flow cytometry detection of B2M expression 7 days post-treatment. **e**, Genome editing activity comparison of CD19 antibody-targeted Cas9-EDV variants packaging *B2M*-targeted Cas9 RNPs. Expression of B2M protein was assessed by flow cytometry 7 days post-treatment in CD19-negative bystander cells. **f,** Direct comparison of genome editing in on-target and bystander cells of the Gag-[3x NES]-[2x p53-NLS]-Cas9 EDV variant. The difference in genome-edited cells between the two cell types is indicated for 1.56 µl and 6.25 µl Cas9-EDV treatment doses. **g,** Treatment of target cells with GS-CA1 does not alter genome editing by Cas9 RNP nucleofection. 293T cells were nucleofected with 50 pmol *B2M*-targeted Cas9 RNPs and either cultured in the presence of 25 nM GS-CA1, 0.05% DMSO, or Opti-MEM. Genome editing was assessed by flow cytometry detection of B2M expression at 3 days post-treatment. Black lines indicate the median of the data set. **h,** Diagram of the Gag-TCL-Cas9 and Gag-TCL-TEV plasmids for producing TEVp-Cas9-EDVs which rely on the TEV protease to release Cas9 from Gag. TCL = TEV protease-cleavable linker. **i,** Western blot analysis of the loss of Gag proteolytic processing in TEVp-Cas9-EDVs, as indicated by the absence of p24 and other Gag proteolytic intermediates. CA= capsid, a component of the gag polypeptide. **j,** Genome editing activity of TEVp-Cas9-EDVs packaging Cas9 RNPs that activate tdTomato expression upon genome editing of a murine neural progenitor cell line harboring a loxP-STOP-loxP-tdTomato reporter[54]. Genome editing was assessed by flow cytometry detection of tdTomato reporter activation 7 days post-treatment. Error bars represent the standard deviation of the mean. N=3 technical replicates were used in all experiments. Four-parameter non-linear regression curves are plotted (**c-f, j**).

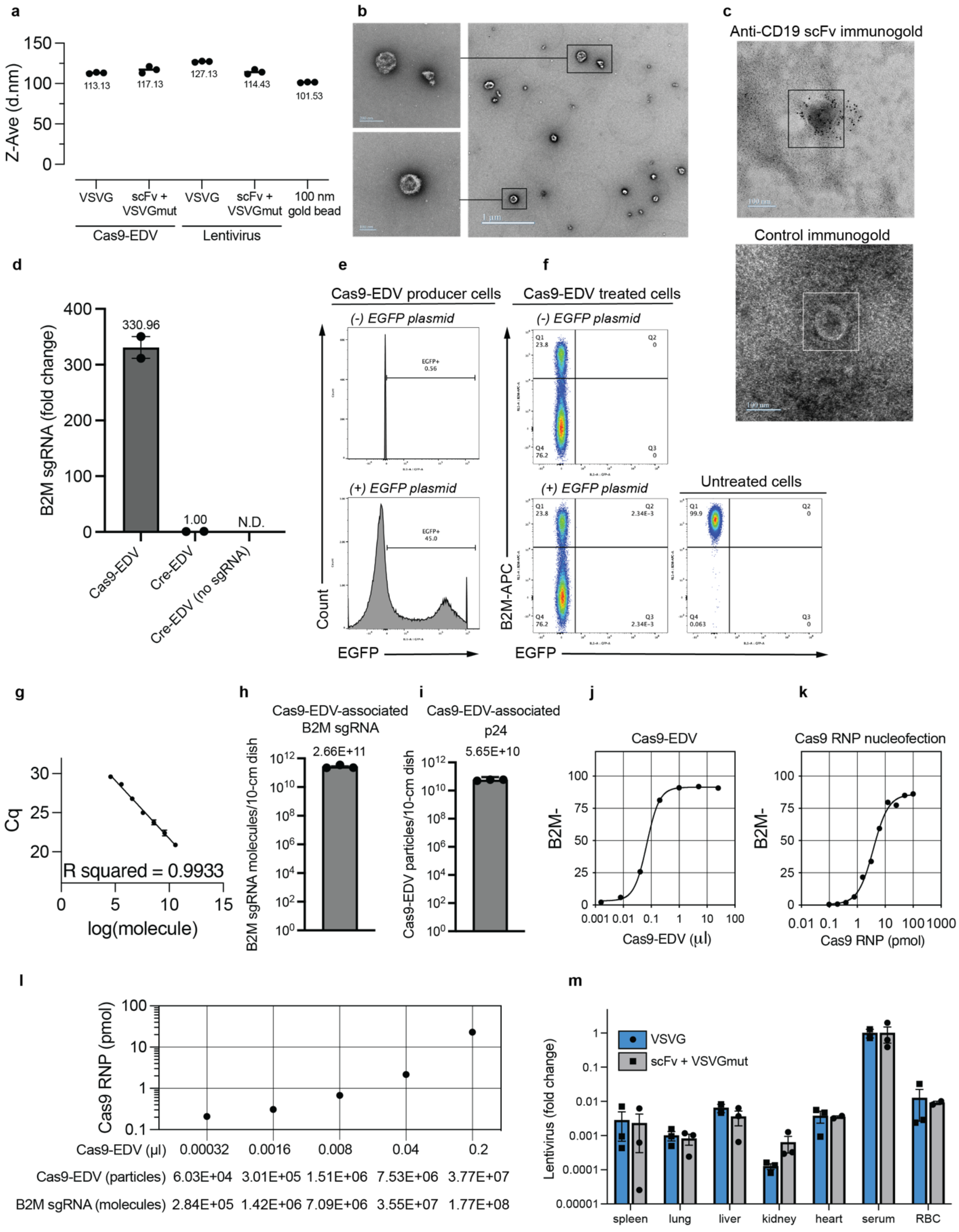

Supplementary Fig. 4. Optimized Cas9-EDV characterization and *in vivo* biodistribution. a, Dynamic light scattering analysis of Cas9-EDV and lentiviral particles. Particles were

pseudotyped with either VSVG or an anti-CD19 scFv+VSVGmut, and the Z-Average (Z-Ave) was measured in nanometers (d.nm). Commercially available 100 nm gold beads were used as a control. N=3 technical replicates. **b,** Transmission electron microscopy (TEM) images of anti-CD19 scFv+VSVGmut-pseudotyped Cas9-EDVs. **c,** Anti-myc immunogold TEM labeling of anti-CD19 scFv 1 targeting molecules on Cas9-EDVs (top). No staining is observed when the anti-CD19 scFv 1 myc tag is swapped for a strep tag (bottom). **d,** Detection of B2M sgRNA in produced Cas9-EDVs and Cre-EDVs. A custom qPCR primer/probe set was used for B2M sgRNA detection, and fold change was calculated by normalizing to the Cre-EDV sample. Cre-EDVs were generated by swapping Cre Recombinase for Cas9 in the Gag-Cas9 V2 plasmid (see **Fig. 2b**). Cre-EDVs (no sgRNA) were produced analogously, but with plasmids lacking the U6-sgRNA expression cassette. See **Supplementary Table 2** for sequences. N.D = not detected. N=2 technical replicates. **e**, To assess the unintended carry-over of plasmid from producer cells to Cas9-EDV treated cells, LentiX cells were transfected to produce Cas9-EDVs either in the absence (top) or the presence (bottom) of 1 μg of an EGFP-expressing plasmid. Flow cytometry was performed on the producer cells three days post-transfection. **f,** Flow cytometry analysis of EGFP-expression in Cas9-EDV treated cells. 293T cells were treated with B2M Cas9-EDVs produced either in the absence (top) or presence (bottom) of an EGFP-expression plasmid. Flow cytometry was performed 6 days post Cas9-EDV treatment, and EGFP and B2M expression were assessed. **g,** Standard curve quantifying synthesized B2M sgRNA via a custom qPCR primer/probe set (R-squared = 0.9933), used to interpolate the number of B2M sgRNAs associated with Cas9-EDVs produced in one 10-cm dish (**h**). N=3 biological replicates of Cas9-EDVs produced in one 10-cm dish and concentrated 30x into 300 μl. **i**, The number of Cas9-EDV particles produced per one 10-cm dish, as estimated by p24 ELISA. N=3 biological replicates. **j,k**, B2M knockout in 293T cells following treatment with the indicated Cas9-EDV volumes (Cas9-EDVs were concentrated 30x) (**j**) or nucleofection with Cas9 RNP complexes (**k**). Flow cytometry was used to detect B2M expression 6 days post-treatment. Four-parameter logistic curves were used to fit the data. **l**, Genome editing comparison between Cas9-EDV treatment volume and Cas9 RNP nucleofection. Data from J and K were used to interpolate how Cas9-EDV treatment volume relates to Cas9 RNP pmol dose. B2M sgRNA molecules and particle numbers are scaled to Cas9-EDV treatment volume (**h** and **i**). **m**, Biodistribution of VSVG (N=3 animals) and anti-CD19 scFv+VSVGmut (N=3 animals) lentivirus. 3E9 lentiviral copies, as normalized by qPCR, were systemically administered to C57BL/6 mice through retro-orbital injections. The indicated tissues/organs were harvested five minutes post-vector administration, RNA was isolated, and qPCR was performed to detect the presence of lentiviral vectors. The quantity of vector is plotted as a fold change relative to the amount of vector in the serum. Each point represents an individual mouse. Two mice received 100 μl PBS as a vehicle control, and no lentiviral vector was detected in any organ, as expected (not shown). Floating numbers (**d, h,** and **i**) indicate the mean. All error bars represent the standard error of the mean. TEM images are representative of two independent experiments (**b, c**).

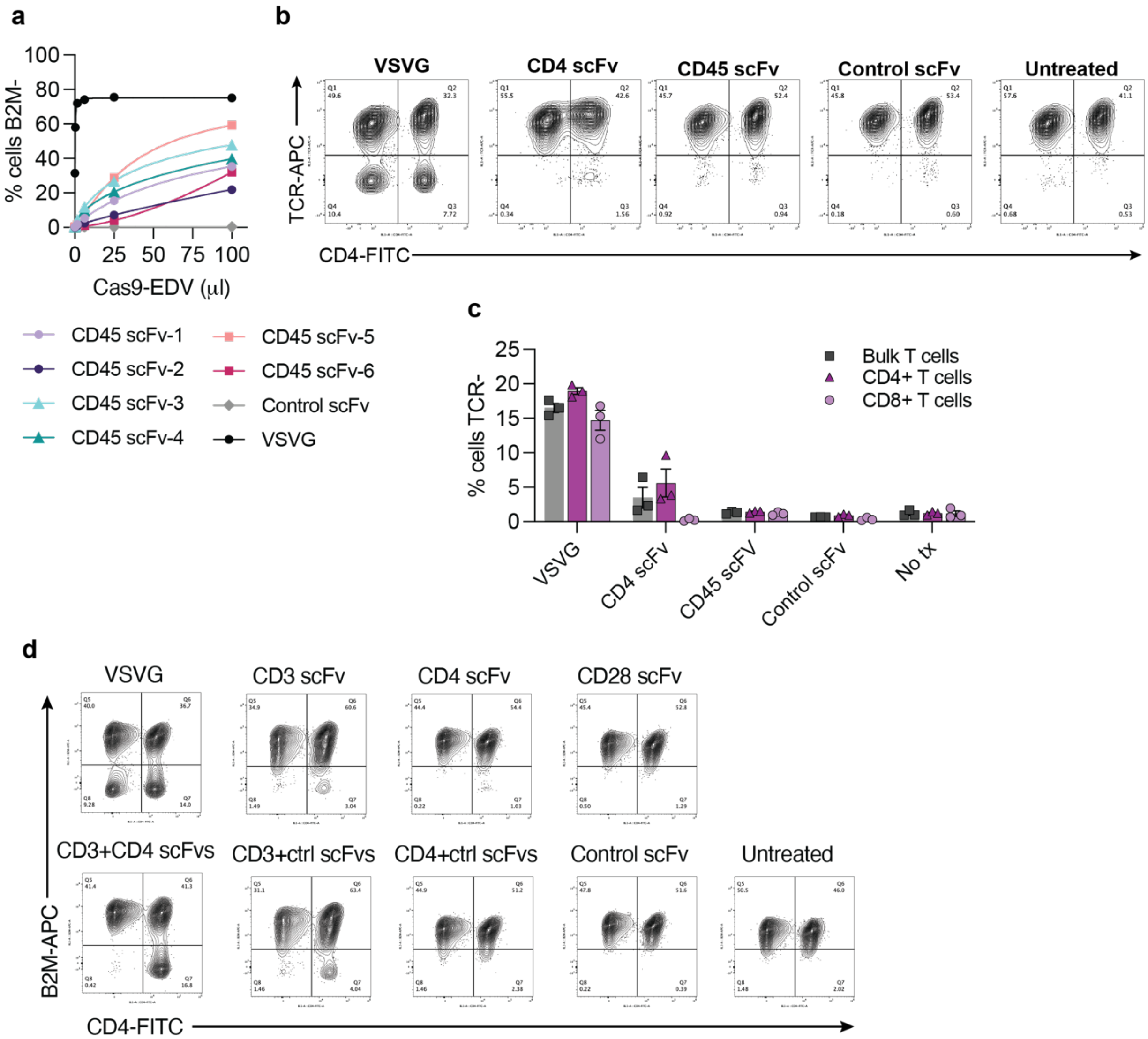

Supplementary Fig. 5. Assessment of T cell-targeting molecules for Cas9-EDV delivery. a, Assessment of human CD45 targeting molecules. A panel of CD45-targeted Cas9-EDVs packaging *B2M*-targeted Cas9 RNPs were produced and concentrated 13.8-fold. 100 µl was used to treat 30k Jurkat cells, and B2M expression was assessed by flow cytometry on day 3. **b**, Assessment of CD4 and CD45-targeted Cas9-EDVs in activated primary human T cells. Cas9-EDVs packaging *TRAC*-targeted Cas9 RNPs were produced, and 1.15×10^7 Cas9-EDVs were used to treat 15k activated T cells. T cell receptor (TCR) loss, indicative of *TRAC* genome editing, was assessed by flow cytometry 5 days post-treatment. Representative plots are shown, and results are quantified in (**c**). CD4 scFv-1 and CD45 scFv-5 targeting molecules were used. **d**, Assessment of multiplexing targeting molecules for delivery to activated primary human T cells. 15k activated T cells were treated with 1.38×10^8 Cas9-EDVs packaging *B2M*-targeted Cas9 RNPs, and flow cytometry analysis of B2M expression was assessed on day 6. Representative flow cytometry plots are shown.CD3 scFv-3, CD4 scFv-2, and CD28 scFv-2 targeting molecules were used. Error bars represent the standard error of the mean. N=3 technical replicates were assessed (**a** and **c**).

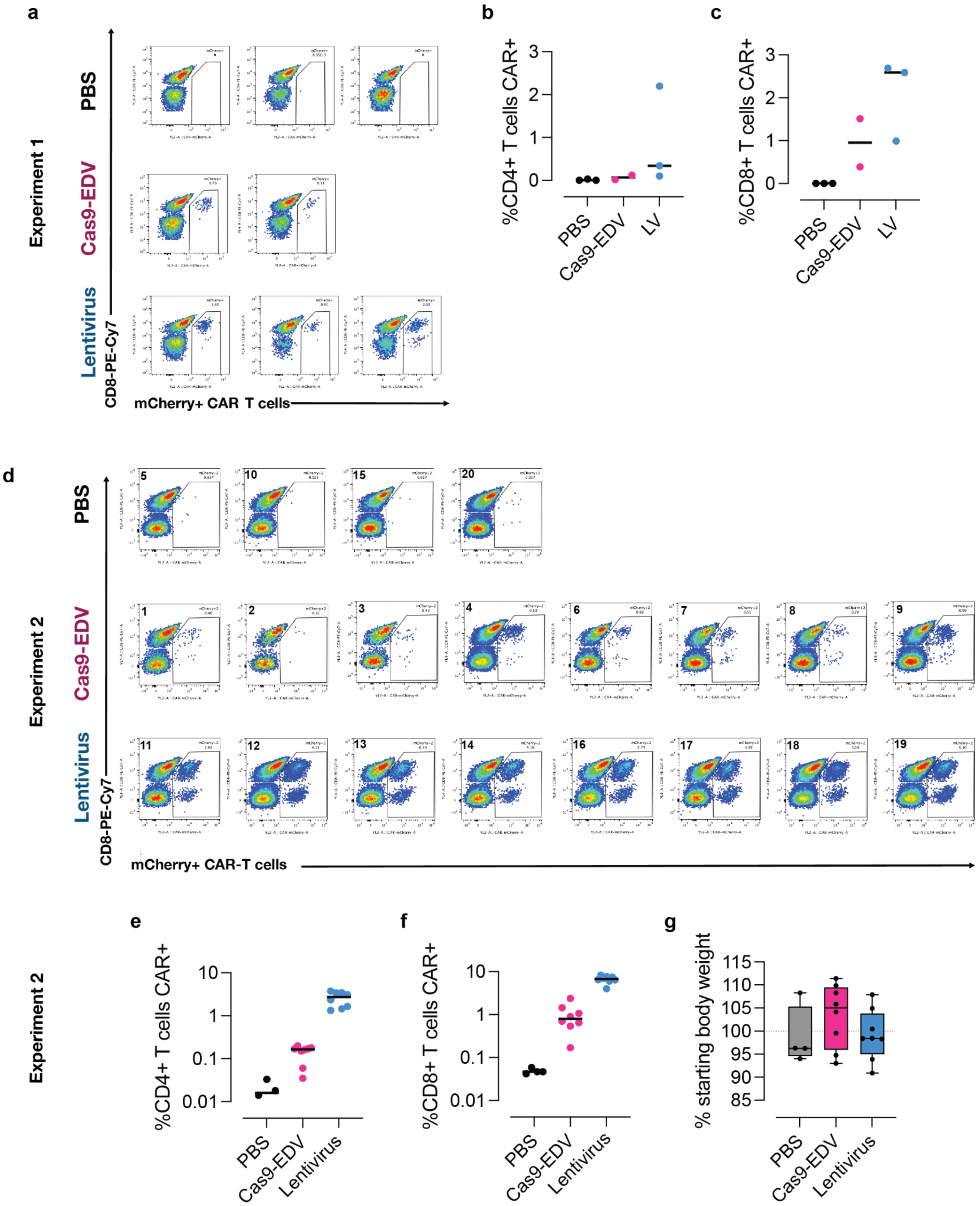
a
Experiment 1
PBS
Cas9-EDV
Lentivirus
CD8-PE-Cy7
mCherry+ CAR T cells
b
%CD4+ T cells CAR+
PBS
Cas9-EDV
LV
c
%CD8+ T cells CAR+
PBS
Cas9-EDV
LV
d
Experiment 2
PBS
Cas9-EDV
Lentivirus
CD8-PE-Cy7
mCherry+ CAR-T cells
e
Experiment 2
%CD4+ T cells CAR+
PBS
Cas9-EDV
Lentivirus
f
%CD8+ T cells CAR+
PBS
Cas9-EDV
Lentivirus
g
% starting body weight
PBS
Cas9-EDV
Lentivirus

Supplementary Fig. 6. Assessment of CAR T generation *in vivo* using T cell-targeted vectors. a-c, Analysis of mCherry+ CAR T cells in spleens from humanized mouse experiment 1. Mice were treated with Cas9-EDV (N=2 animals), lentivirus (N=3 animals), or PBS, and flow cytometry analysis was performed 10 days post-treatment. Flow cytometry quantification of CD4+ (**b**) and CD8+ CAR T cells (**c**). **d-f**, Analysis of mCherry+ CAR T cells in spleens from humanized mouse experiment 2. Mice were treated with Cas9-EDV (N=8 animals), lentivirus (N=8 animals), or PBS (N=4 animals), and flow cytometry analysis was performed 10 days post-treatment. Upper left quadrant numbers are mouse identifiers. Flow cytometry quantification of CD4+ (**e**) and CD8+ CAR T cells (**f**). **g**, Quantification of body weight change in humanized mice from experiment 2. The box extends from the 25th to 75th percentile, with the line in the center of the box indicating the median. Whiskers indicate minimum and maximum values, and the dotted line indicates 100% starting body weight. PBS = phosphate-buffered saline. Black lines indicate the median (**b, c, e-g**).

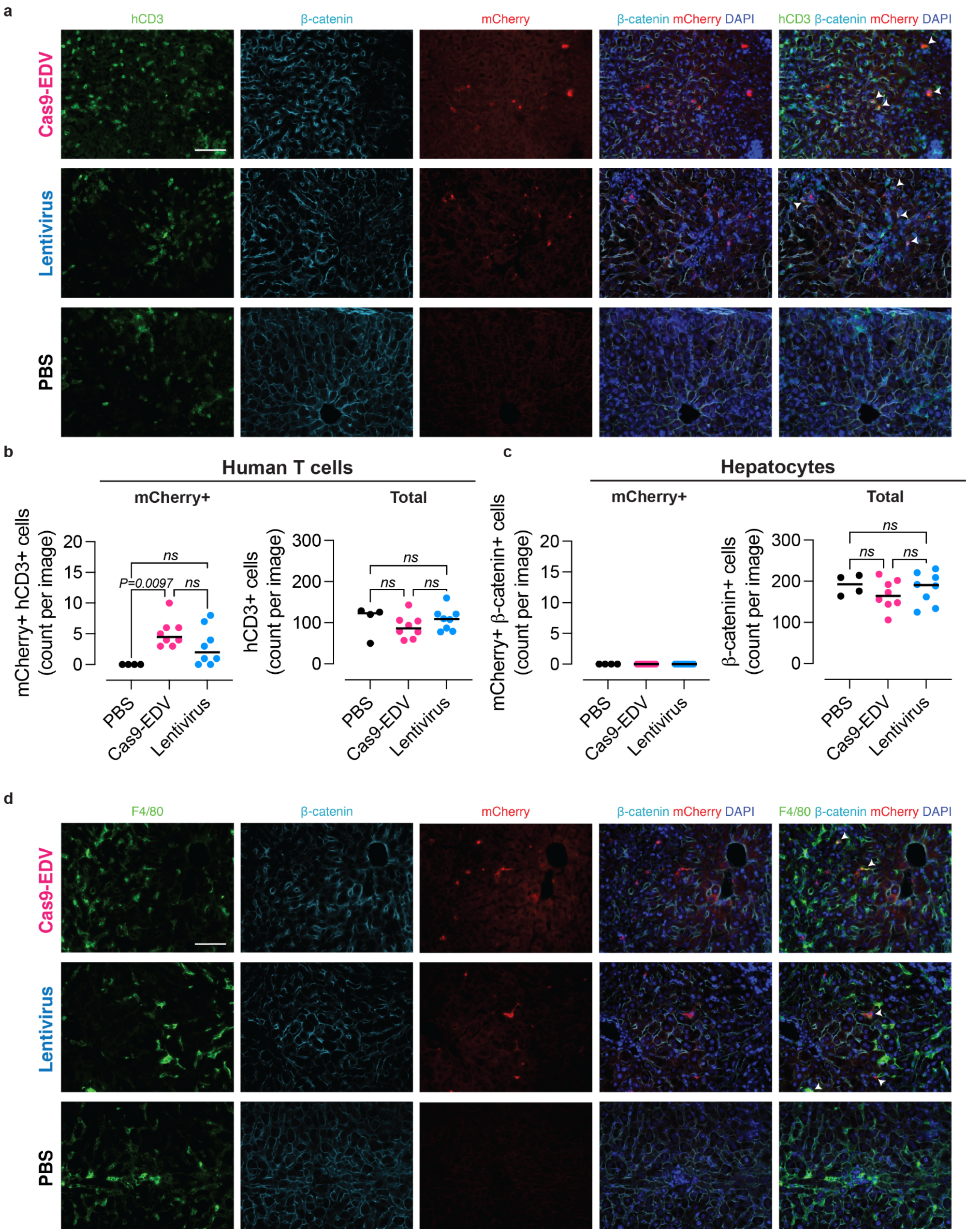
a
hCD3
β-catenin
mCherry
β-catenin mCherry DAPI
hCD3 β-catenin mCherry DAPI
Cas9-EDV
Lentivirus
PBS
b
Human T cells
mCherry+
Total
mCherry+ hCD3+ cells (count per image)
hCD3+ cells (count per image)
P=0.0097
ns
PBS
Cas9-EDV
Lentivirus
c
Hepatocytes
mCherry+
Total
mCherry+ β-catenin+ cells (count per image)
β-catenin+ cells (count per image)
d
F4/80
β-catenin
mCherry
β-catenin mCherry DAPI
F4/80 β-catenin mCherry DAPI

Supplementary Fig. 7. Analysis of delivery to off-target cells *in vivo.* Immunofluorescence (IF) imaging of livers isolated from PBMC-humanized mice 10 days post systemic administration of T-cell targeted Cas9-EDV (N=8 animals) and lentivirus (N=8 animals). **a**, mCherry expression (red), a marker of transduction, co-localizes with human CD3+ T cells (hCD3) (green) but not β-catenin+ hepatocytes (teal). **b,c,** Quantification of IF images. While mCherry+ hCD3+ T cells are detected (**b**), no mCherry+ β-catenin+ hepatocytes are observed (**c**). Each point represents the quantification of a representative IF image from an individual mouse. P values were calculated by means of a Tukey multiple comparison test after ordinary one-way ANOVA. $^{**}P < 0.01$. ns = not significant ($P > 0.05$). **d,** mCherry+ F4/80+ (green) phagocytic cells are also detected. Representative confocal microscopy images are shown (a and **d**). The scale bar indicates 65 μm. Immunofluorescence images are representative of two independent experiments (**a,d**).

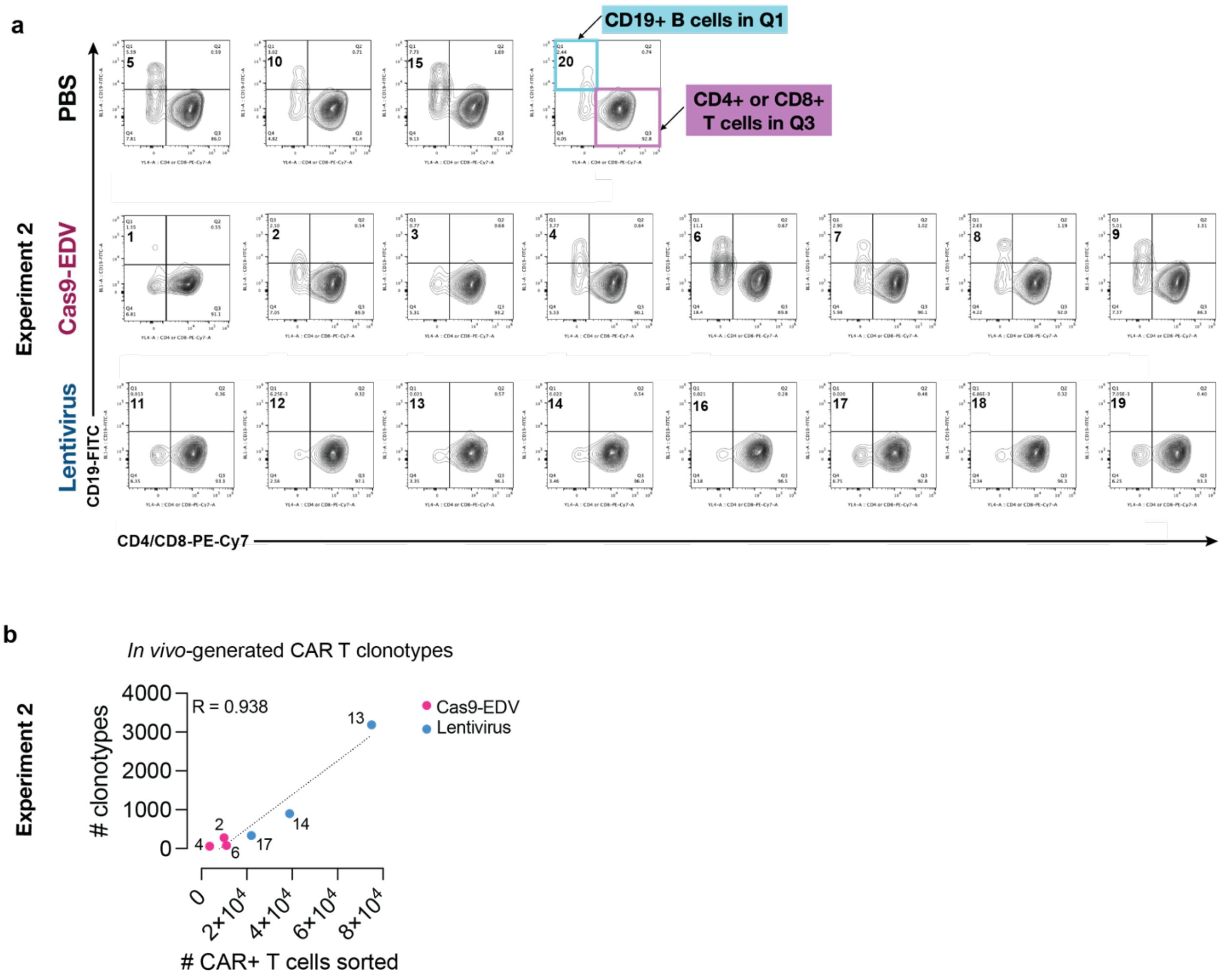

Supplementary Fig. 8. Assessment of CD19+ B cells and CAR T cell receptor clonality following *in vivo* cellular engineering. a, Flow cytometry analysis of CD19+ B cells in Cas9-EDV- or lentivirus-treated mice 10 days post-administration. Human CD45+ cells were isolated from humanized mouse spleens prior to flow cytometry analysis. Upper left quadrant numbers are mouse identifiers. **b,** Correlation between the number of *in vivo*-generated CAR T clonotypes versus the number of CAR T cells sorted (Pearson's correlation coefficient, R = 0.938). Point labels indicate the unique mouse analyzed.

Supplementary Information References

54. Staahl, B. T. et al. Efficient genome editing in the mouse brain by local delivery of engineered Cas9 ribonucleoprotein complexes. *Nat. Biotechnol.* **35**, 431–434 (2017).
