# Supplementary material for: In vivo human T cell engineering with enveloped delivery vehicles
Source: Nat Biotechnol. 2024 Jan 11;42(11):1684–92. doi: 10.1038/s41587-023-02085-z (PMC11236958; doi:10.1038/s41587-023-02085-z)
Supplement: Supplementary file 2 — Reporting Summary [file 41587_2023_2085_MOESM2_ESM.pdf]

nature portfolio

Corresponding author(s): Jennifer Doudna

Last updated by author(s): Oct 6, 2023

Reporting Summary

Nature Portfolio wishes to improve the reproducibility of the work that we publish. This form provides structure for consistency and transparency in reporting. For further information on Nature Portfolio policies, see our Editorial Policies and the Editorial Policy Checklist.

Statistics

For all statistical analyses, confirm that the following items are present in the figure legend, table legend, main text, or Methods section.

n/a	Confirmed	
☐	☒	The exact sample size (*n*) for each experimental group/condition, given as a discrete number and unit of measurement
☐	☒	A statement on whether measurements were taken from distinct samples or whether the same sample was measured repeatedly
☐	☒	The statistical test(s) used AND whether they are one- or two-sided<br>*Only common tests should be described solely by name; describe more complex techniques in the Methods section.*
☒	☐	A description of all covariates tested
☐	☒	A description of any assumptions or corrections, such as tests of normality and adjustment for multiple comparisons
☐	☒	A full description of the statistical parameters including central tendency (e.g. means) or other basic estimates (e.g. regression coefficient) AND variation (e.g. standard deviation) or associated estimates of uncertainty (e.g. confidence intervals)
☐	☒	For null hypothesis testing, the test statistic (e.g. F, t, r) with confidence intervals, effect sizes, degrees of freedom and P value noted<br>*Give P values as exact values whenever suitable.*
☒	☐	For Bayesian analysis, information on the choice of priors and Markov chain Monte Carlo settings
☒	☐	For hierarchical and complex designs, identification of the appropriate level for tests and full reporting of outcomes
☐	☒	Estimates of effect sizes (e.g. Cohen's d, Pearson's r), indicating how they were calculated

Our web collection on statistics for biologists contains articles on many of the points above.

Software and code

Policy information about availability of computer code

Data collection	The Attune NxT Software was used to collect flow cytometry data. ECHOPro was used to collect microscopy images. QuantStudio 6 was used to collect RT-qPCR data.
Data analysis	FlowJo v10 10.7.1, CRISPResso2, Geneious Prime 2023, and Cogent NGS Immune Profiler Software v1.5).<br>All original code used in this study has been deposited online and is publicly available on GitHub (DOI: 10.5281/zenodo.8417852)

For manuscripts utilizing custom algorithms or software that are central to the research but not yet described in published literature, software must be made available to editors and reviewers. We strongly encourage code deposition in a community repository (e.g. GitHub). See the Nature Portfolio guidelines for submitting code & software for further information.

Data

Policy information about availability of data

All manuscripts must include a data availability statement. This statement should provide the following information, where applicable:

- Accession codes, unique identifiers, or web links for publicly available datasets
- A description of any restrictions on data availability
- For clinical datasets or third party data, please ensure that the statement adheres to our policy

Sequencing data have been deposited in the National Institutes of Health NCBI SRA (BioProject PRJNA1023251) and GEO (accession number GSE235643) repositories. Flow cytometry raw data files are available upon request. All other data are available in the main text or the supplementary materials. Plasmids

generated in this study are available on Addgene.

Research involving human participants, their data, or biological material

Policy information about studies with human participants or human data. See also policy information about sex, gender (identity/presentation), and sexual orientation and race, ethnicity and racism.

Reporting on sex and gender	N/A
Reporting on race, ethnicity, or other socially relevant groupings	N/A
Population characteristics	N/A
Recruitment	N/A
Ethics oversight	N/A

Note that full information on the approval of the study protocol must also be provided in the manuscript.

Field-specific reporting

Please select the one below that is the best fit for your research. If you are not sure, read the appropriate sections before making your selection.

☒ Life sciences ☐ Behavioural & social sciences ☐ Ecological, evolutionary & environmental sciences

For a reference copy of the document with all sections, see nature.com/documents/nr-reporting-summary-flat.pdf

Life sciences study design

All studies must disclose on these points even when the disclosure is negative.

Sample size	Sample sizes were determined based on preliminary experiments and/or experimental design used in related studies.
Data exclusions	One sample was excluded in Fig. 4F due to failing sequencing.
Replication	All experiments were replicated at least twice, with one representative experiment shown. Experiment 1 and 2 replicates are shown for humanized mouse expeirments (Fig. 4).
Randomization	In animal experiments, mice were randomized by cage and littermates.
Blinding	Blinding was not relevant to the experiments due to the experimental settings described in methods and legends.

Reporting for specific materials, systems and methods

We require information from authors about some types of materials, experimental systems and methods used in many studies. Here, indicate whether each material, system or method listed is relevant to your study. If you are not sure if a list item applies to your research, read the appropriate section before selecting a response.

Materials & experimental systems

n/a	Involved in the study	
☐	☒	Antibodies
☐	☒	Eukaryotic cell lines
☒	☐	Palaeontology and archaeology
☐	☒	Animals and other organisms
☒	☐	Clinical data
☒	☐	Dual use research of concern
☒	☐	Plants

Methods

n/a	Involved in the study	
☒	☐	ChIP-seq
☐	☒	Flow cytometry
☒	☐	MRI-based neuroimaging

Antibodies

Antibodies used

anti-FLAG,Sigma-Aldrich,M2,"F1804, RRID:AB_262044",WB (1:1000),https://www.sigmaaldrich.com/US/en/product/sigma/f1804
anti-HIV1 p24,abcam,"ab9071, RRID:AB_306981",WB (1:1000),https://www.abcam.com/products/primary-antibodies/hiv1-p24-antibody-ab63913
anti-mouse IgG (H+L) Alexa Fluor™ 488,Thermo Fisher Scientific,"A28175, RRID:AB_2536161",WB (1:10000),https://www.thermofisher.com/antibody/product/Goat-anti-Mouse-IgG-H-L-Secondary-Antibody-Recombinant-Polyclonal/A28175
anti-rabbit IgG (H+L) Alexa Fluor™ 647,Thermo Fisher Scientific,"A-31573, RRID:AB_2536183",WB (1:10000),https://www.thermofisher.com/antibody/product/Donkey-anti-Rabbit-IgG-H-L-Highly-Cross-Adsorbed-Secondary-Antibody-Polyclonal/A-31573
anti-human CD4-FITC,Biolegend,RPA-T4,"300538, RRID:AB_2562052",Flow Cytometry (1:100),https://www.biolegend.com/en-us/products/fitc-anti-human-cd4-antibody-825
anti-human CD4-PE-Cyanine7,Biolegend,RPA-T4,"300512, RRID:AB_314080",Flow Cytometry (1:100),https://www.biolegend.com/fr-fr/cell-health/pe-cyanine7-anti-human-cd4-antibody-829
anti-human CD8-PE-Cyanine7,BD Biosciences,RPA-T8,"557746, RRID:AB_396852",Flow Cytometry (1:100),https://www.bdbiosciences.com/en-us/products/reagents/flow-cytometry-reagents/research-reagents/single-color-antibodies-ruo/pe-cy-7-mouse-anti-human-cd8.557746
anti-human CD19-FITC,Biolegend,HIB19,"302206, RRID:AB_314236",Flow Cytometry (1:100),https://www.biolegend.com/en-us/products/fitc-anti-human-cd19-antibody-717
anti-human CD3-FITC,Biolegend,OKT3,"317306, RRID:AB_571907",Flow Cytometry (1:100),https://www.biolegend.com/en-ie/products/fitc-anti-human-cd3-antibody-3644
anti-human B2M-APC,Biolegend,2M2,"316312, RRID:AB_10641281",Flow Cytometry (1:100),https://www.biolegend.com/en-us/soluble-mhc/apc-anti-human-beta2-microglobulin-antibody-6910
anti-human B2M-PE,Biolegend,2M2,"316306, RRID:AB_492839",Flow Cytometry (1:100),https://www.biolegend.com/de-de/cell-health/pe-anti-human-beta2-microglobulin-antibody-3080
anti-human CD28-PE,Biolegend,CD28.2,"302907, RRID:AB_314309",Flow Cytometry (1:100),https://www.biolegend.com/nl-be/products/pe-anti-human-cd28-antibody-630
anti-human CD20-PE,Biolegend,2H7,"302306, RRID:AB_314254",Flow Cytometry (1:100),https://www.biolegend.com/nl-nl/soluble-mhc/pe-anti-human-cd20-antibody-559
anti-human CD19-PE,Biolegend,H1B19,"302208, RRID:AB_314238",Flow Cytometry (1:100),https://www.biolegend.com/en-ie/products/pe-anti-human-cd19-antibody-719
anti-human CD25-APC,Biolegend,BC96,"302610, RRID:AB_314280",Flow Cytometry (1:100),https://www.biolegend.com/en-us/products/apc-anti-human-cd25-antibody-614
anti-β-catenin,Thermo Fisher Scientific,15B8,"14-2567-82, RRID:AB_1724004",IHC (1:100),https://www.thermofisher.com/antibody/product/beta-Catenin-Antibody-clone-15B8-Monoclonal/14-2567-82
anti-F4/80,Novus,CI-A3-1,"NB600-404, RRID:AB_10003219",IHC (1:100),https://www.novusbio.com/products/f4-80-antibody-ci-a3-1_nb600-404
anti-human CD3,Abcam,"ab5690, RRID:AB_305055",IHC (1:100),https://www.abcam.com/en-ee/products/primary-antibodies/anti-cd3-antibody-ab5690
anti-mouse IgG (H+L) AlexaFluor™ 647,Thermo Fisher Scientific,"A21236, RRID:AB_2535805",IHC (1:200),https://www.thermofisher.com/antibody/product/Goat-anti-Mouse-IgG-H-L-Highly-Cross-Adsorbed-Secondary-Antibody-Polyclonal/A-21236
anti-Rat IgG (H+L) AlexaFluor™ 488 ,Thermo Fisher Scientific,"A11006, RRID:AB_2534074",IHC (1:200),https://www.thermofisher.com/antibody/product/Goat-anti-Rat-IgG-H-L-Cross-Adsorbed-Secondary-Antibody-Polyclonal/A-11006
anti-rabbit IgG (H+L) AlexaFluor™ 488 ,Thermo Fisher Scientific,"A11034, RRID:AB_2576217",IHC (1:200),https://www.thermofisher.com/antibody/product/Goat-anti-Rabbit-IgG-H-L-Highly-Cross-Adsorbed-Secondary-Antibody-Polyclonal/A-11034
anti-myc,Abcam,9E10,"ab32, RRID:AB_303599",,https://www.abcam.com/products/primary-antibodies/myc-tag-antibody-9e10-ab32

Validation

Antibodies were validated for the specific application by the manufacturers, and validation data are available on the manufacturer's website, as linked above.

Eukaryotic cell lines

Policy information about cell lines and Sex and Gender in Research

Cell line source(s)

Lenti-X and HEK293T cells are obtained from the UC Berkeley Cell Culture Facility. Human peripheral blood mononuclear cells were obtained from AllCells.

Authentication

Authentication of Lenti-X and HEK293T cells was performed using STR by the UC Berkeley Cell Culture Facility. Human PBMCs were authenticated by flow cytometry.

Mycoplasma contamination

All cell lines/cells used here tested negative for mycoplasma contamination.

Commonly misidentified lines
(See ICLAC register)

No commonly misidentified line was used in the study.

Animals and other research organisms

Policy information about studies involving animals; ARRIVE guidelines recommended for reporting animal research, and Sex and Gender in Research

Laboratory animals

Human PBMC-engrafted NSG™ mice (745557) and C57BL/6 mice (000664) were obtained from Jackson Laboratory. PBMC-engrafted

Laboratory animals	mice were between 6 and 8 weeks old at the time of treatment, and C57BL/6 mice were between 9 and 10 weeks old at the time of treatment.
Wild animals	No wild animals were used in the study.
Reporting on sex	The applicability of the findings is not sex-specific; no sex-based analysis was performed.
Field-collected samples	No field-collected samples were used in the study.
Ethics oversight	All procedures were approved by the UC Berkeley Animal Care & Use Committee.

Note that full information on the approval of the study protocol must also be provided in the manuscript.

Plants

Seed stocks	*Report on the source of all seed stocks or other plant material used. If applicable, state the seed stock centre and catalogue number. If plant specimens were collected from the field, describe the collection location, date and sampling procedures.*
Novel plant genotypes	*Describe the methods by which all novel plant genotypes were produced. This includes those generated by transgenic approaches, gene editing, chemical/radiation-based mutagenesis and hybridization. For transgenic lines, describe the transformation method, the number of independent lines analyzed and the generation upon which experiments were performed. For gene-edited lines, describe the editor used, the endogenous sequence targeted for editing, the targeting guide RNA sequence (if applicable) and how the editor was applied.*
Authentication	*Describe any authentication procedures for each seed stock used or novel genotype generated. Describe any experiments used to assess the effect of a mutation and, where applicable, how potential secondary effects (e.g. second site T-DNA insertions, mosiacism, off-target gene editing) were examined.*

Flow Cytometry

Plots

Confirm that:

☒ The axis labels state the marker and fluorochrome used (e.g. CD4-FITC).

☒ The axis scales are clearly visible. Include numbers along axes only for bottom left plot of group (a 'group' is an analysis of identical markers).

☒ All plots are contour plots with outliers or pseudocolor plots.

☒ A numerical value for number of cells or percentage (with statistics) is provided.

Methodology

Sample preparation	Samples were prepared as described in Methods.
Instrument	Attune NxT flow cytometer with 96-well autosampler was used.
Software	FlowJo v10 10.7.1 was used to analyze data.
Cell population abundance	Gated cell population abundance is presented on the original flow plots or in summary graphs.
Gating strategy	Gating strategy is shown in Fig. 1, 4 and Fig. S4, S5, S6, S8.

☒ Tick this box to confirm that a figure exemplifying the gating strategy is provided in the Supplementary Information.
